# Mutations Elevate an Underground Pathway to a Physiologically Relevant Protopathway

**DOI:** 10.1093/molbev/msaf193

**Published:** 2025-08-07

**Authors:** Karl A Widney, Lauren C Phillips, Leo M Rusch, Shelley D Copley

**Affiliations:** Department of Biochemistry, University of Colorado Boulder, Boulder, CO 80309, USA; Cooperative Institute for Research in Environmental Sciences, University of Colorado, Boulder, CO 80205, USA; Cooperative Institute for Research in Environmental Sciences, University of Colorado, Boulder, CO 80205, USA; Department of Molecular, Cellular and Developmental Biology, University of Colorado Boulder, Boulder, CO 80309, USA; Cooperative Institute for Research in Environmental Sciences, University of Colorado, Boulder, CO 80205, USA; Department of Molecular, Cellular and Developmental Biology, University of Colorado Boulder, Boulder, CO 80309, USA; Cooperative Institute for Research in Environmental Sciences, University of Colorado, Boulder, CO 80205, USA; Department of Molecular, Cellular and Developmental Biology, University of Colorado Boulder, Boulder, CO 80309, USA

**Keywords:** mutations, PLP, evolution, enzyme promiscuity, metabolic pathway, protopathway

## Abstract

Underground metabolic pathways—leaks in the metabolic network caused by promiscuous enzyme activities and nonenzymatic transformations—can provide the starting point for emergence of novel protopathways if a mutation or environmental change increases flux to a physiologically significant level. This early stage in pathway evolution, in which promiscuous enzymes are still serving their native functions and proper regulation has not yet emerged, is typically hidden from our view. We previously used laboratory evolution to evolve a novel four-step protopathway in Δ*pdxB E. coli*, which lacks an enzyme required for synthesis of pyridoxal 5′-phosphate (PLP). By sequencing population genomic DNA from samples archived during the evolution experiment, we have identified mutations that rose and fell in abundance in the population leading to JK1, the dominant clone after 150 population doublings. We have identified the order in which the four mutations arose in JK1 and the physiological effect of each mutation. The first mutation increases the rate of PLP synthesis. The second mutation did not impact PLP synthesis but rather created a cheater that thrived in the population by scavenging nutrients released from the fragile parental cells. Notably, the dominant lineages at the end of the experiment all derived from this cheater strain. The third mutation in JK1 destroyed a PLP phosphatase, which preserves precious PLP. Finally, the fourth mutation improved growth in glucose after the PLP synthesis problem had been solved. Together, these mutations resulted in restoration of PLP synthesis and a 32-fold increase in growth rate.

## Introduction

Metabolic networks in extant organisms are complex, efficient, and exquisitely regulated. Yet nothing in biology is perfect. Promiscuous enzyme reactions and nonenzymatic decomposition reactions cause innumerable leaks in metabolic pathways. The term “underground metabolism” was coined to describe reactions and even multistep pathways that arise from leaks in the metabolic network (D'Ari and Casadesus 1998). Underground reactions can decrease the efficiency of metabolic pathways, generate useless waste products, and even produce compounds that are toxic (Bommer et al. 2020). If production of an unintended product by an underground reaction rises to a level that impairs fitness, natural selection favors mutants that minimize the problem. One solution is to sculpt the active site of a promiscuous enzyme to decrease production of a problematic molecule. Another is to recruit a “metabolite repair” enzyme or pathway to detoxify the problematic molecule and, in many cases, to return it to the metabolic network (Bommer et al. 2020). For example, the metabolite repair enzyme oxaloacetate tautomerase converts toxic enol-oxaloacetate produced by succinate dehydrogenase to the metabolically useful keto form (Zmuda et al. 2024).

Although underground reactions are normally physiologically irrelevant because natural selection has minimized fluxes or resulted in metabolite repair strategies, they do provide opportunities for innovation. Even inefficient promiscuous enzymes can accelerate chemical reactions by orders of magnitude (O'Brien and Herschlag 1999; Lassila and Herschlag 2008; Olguin et al. 2008), providing a useful starting point for the evolution of new enzymes. Further, many enzymes act on a large number of noncanonical substrates. A screen of >200 phosphatases in the haloalkanoate dehalogenase (HAD) superfamily from 86 species against 167 potential substrates showed that the majority acted on more than 5 substrates; 50 enzymes turned over more than 41 and as many as 143 substrates (Huang et al. 2015). Other studies have demonstrated widespread promiscuity in metallo-β-lactamases (Baier and Tokuriki 2014), flavin-dependent halogenases (Fisher et al. 2019), sulfate monoester and phosphotriester hydrolases (Colin et al. 2015), and the DUF849 PFAM family (Bastard et al. 2014). Thus, the hundreds or thousands of enzymes within any proteome harbor a vast reservoir of catalytic potential.

Much work by enzymologists, metabolic engineers, and synthetic biologists has explored the potential for evolution of new specialized enzymes starting from promiscuous enzymes. However, the potential for metabolic innovation provided by promiscuous enzymes extends beyond evolution of individual new enzymes. Recruitment of one or more promiscuous reactions in an underground pathway could jump-start the evolution of a new metabolic pathway (D'Ari and Casadesus 1998; Notebaart et al. 2018; Guzman et al. 2019).

Bioinformatic evidence suggests that metabolic pathways evolve by patching together enzymes that have a promiscuous ability to catalyze newly important reactions, followed by gene duplication and divergence to provide enzymes specialized for their new functions (Teichmann et al. 2001; Gerlt et al. 2005; Roodveldt and Tawfik 2005; Burroughs et al. 2006; Bergthorsson et al. 2007). However, the retrospective view provided by bioinformatics reveals little about the *process* by which new pathways emerge from underground pathways. The suite of promiscuous activities available within a proteome and the mutations that allowed an initial “protopathway” to emerge are largely lost in time. We define “protopathways” as the earliest stage in the evolution of novel pathways. At this stage, promiscuous enzymes that have been recruited to serve new functions are still serving their native functions, and proper regulation has not emerged. Protopathways likely follow the course of underground pathways (D'Ari and Casadesus 1998) but are different because a mutation or environmental change has made flux through the pathway physiologically relevant.

**Fig. 1.**
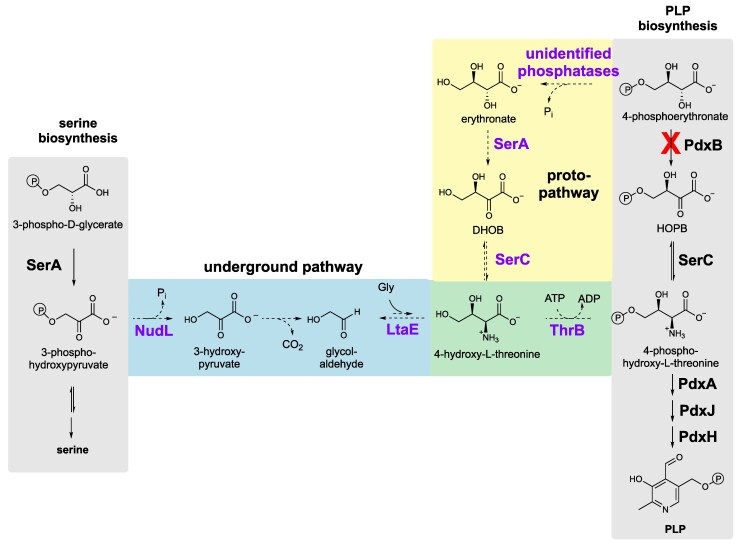
Promiscuous enzyme activities can be patched together to generate novel pathways for production of 4-phosphohydroxy-L-threonine. The four-step underground pathway can restore the growth of Δ*pdxB E. coli* if either NudL or ThrB is overexpressed. The four-step protopathway restores PLP synthesis in the evolved Δ*pdxB E. coli* strain JK1. Blue, underground pathway; yellow, protopathway; green, a step common to the underground pathway and the protopathway; purple, promiscuous enzyme activities. DHOB, (*3R*)-3,4-dihydroxy-2-oxobutanoate; HOPB, (*3R*)-3-hydroxy-2-oxo-4-phosphooxybutanoate; SerA, 3-phosphoglycerate dehydrogenase; SerC, phosphoserine/phosphohydroxythreonine transaminase; ThrB, homoserine kinase; PdxB, 4PE dehydrogenase; PdxA, 4-phosphohydroxy-L-threonine dehydrogenase; PdxJ, pyridoxine 5′-phosphate synthase; PdxH, pyridoxine/pyridoxamine 5′-phosphate oxidase. Co-substrates for several reactions are omitted for clarity.

We previously unmasked an underground pathway in Δ*pdxB E. coli* (Fig. 1) that restores synthesis of the essential cofactor pyridoxal 5′-phosphate (PLP) by producing 4-phosphohydroxy-L-threonine, an intermediate downstream of the break in the PLP synthesis pathway caused by loss of PdxB (4-phosphoerythronate dehydrogenase). Thus, this model system mimics the general situation in which an organism needs to produce a particular metabolite for either a catabolic or anabolic purpose. Sufficient 4-phosphohydroxy-L-threonine can be produced in Δ*pdxB E. coli* by overexpression of either NudL (a putative CoA pyrophosphorylase) or ThrB (homoserine kinase) on a high-copy plasmid (Kim et al. 2010). Overexpression of NudL pushes material into the pathway due to its promiscuous ability to hydrolyze 3-phosphohydroxypyruate. Overexpression of ThrB pulls material through the pathway due to a surprisingly robust promiscuous ability to phosphorylate 4-hydroxythreonine (*k*_cat_/*K_M_* = 4.8 × 10^3 ^M^−1 ^s^−1^). Without overexpression of NudL or ThrB, flux through this underground pathway is physiologically irrelevant because the canonical PLP synthesis is far more efficient. We wondered whether mutations in Δ*pdxB E. coli* could elevate this underground pathway to physiological significance. We previously evolved multiple replicates of Δ*pdxB E. coli* in M9/glucose, conditions under which PLP synthesis is essential (Kim et al. 2019). The Δ*pdxB* strain initially grew extremely slowly. However, within 150 population doublings, we obtained strains that grew at up to 74% the rate of wild-type *E. coli.* Strain JK1 generates wild-type levels of PLP (Kim et al. 2019) by patching together a novel protopathway comprised of four steps catalyzed by promiscuous enzymes that shares only a single reaction with the previously discovered underground pathway (Fig. 1). This finding suggests that multiple underground pathways may exist for formation of a target metabolite in a bacterial proteome.

Strain JK1 had accumulated four mutations (Kim et al. 2019). In the present study, we analyzed population genomic DNA (gDNA) from samples archived at intervals during the evolution of JK1 to identify the sequence of mutations that led to JK1 as well as the overall population dynamics. We reconstructed all possible evolutionary intermediates between Δ*pdxB E. coli* and strain JK1 and characterized their rates of growth and PLP accumulation. Multiple trajectories could have led to JK1. The actual trajectory began with a mutation in *gapA*, which encodes glyceraldehyde 3-phosphate dehydrogenase (GAPDH). The impaired activity of GAPDH in glycolysis decreases the concentration of serine in the cells and improves the ability of SerA, which is subject to feedback inhibition by serine, to function in the protopathway. The second mutation caused loss of RpoS, the master regulator of the stress response. This mutation does not improve PLP synthesis but rather allows the gapA* rpoS* strain to avoid a stress-induced slowdown in growth due to the stress of nutrient limitation and, in the context of the population, to grow faster by taking advantage of nutrients released by lysis of the fragile parental cells. The third mutation, a large deletion that destroys the broad-specificity phosphatase YbhA (Kuznetsova et al. 2006), prevents degradation of PLP. The final point mutation in *rpoC*, which encodes the β′ subunit of RNA polymerase, improves growth in minimal medium, a finding consistent with results of previous studies (Conrad et al. 2010; Cheng et al. 2014; Sandberg et al. 2014).

## Results and Discussion

### Complex Population Dynamics Preceded the Emergence of JK1

**Fig. 2.**
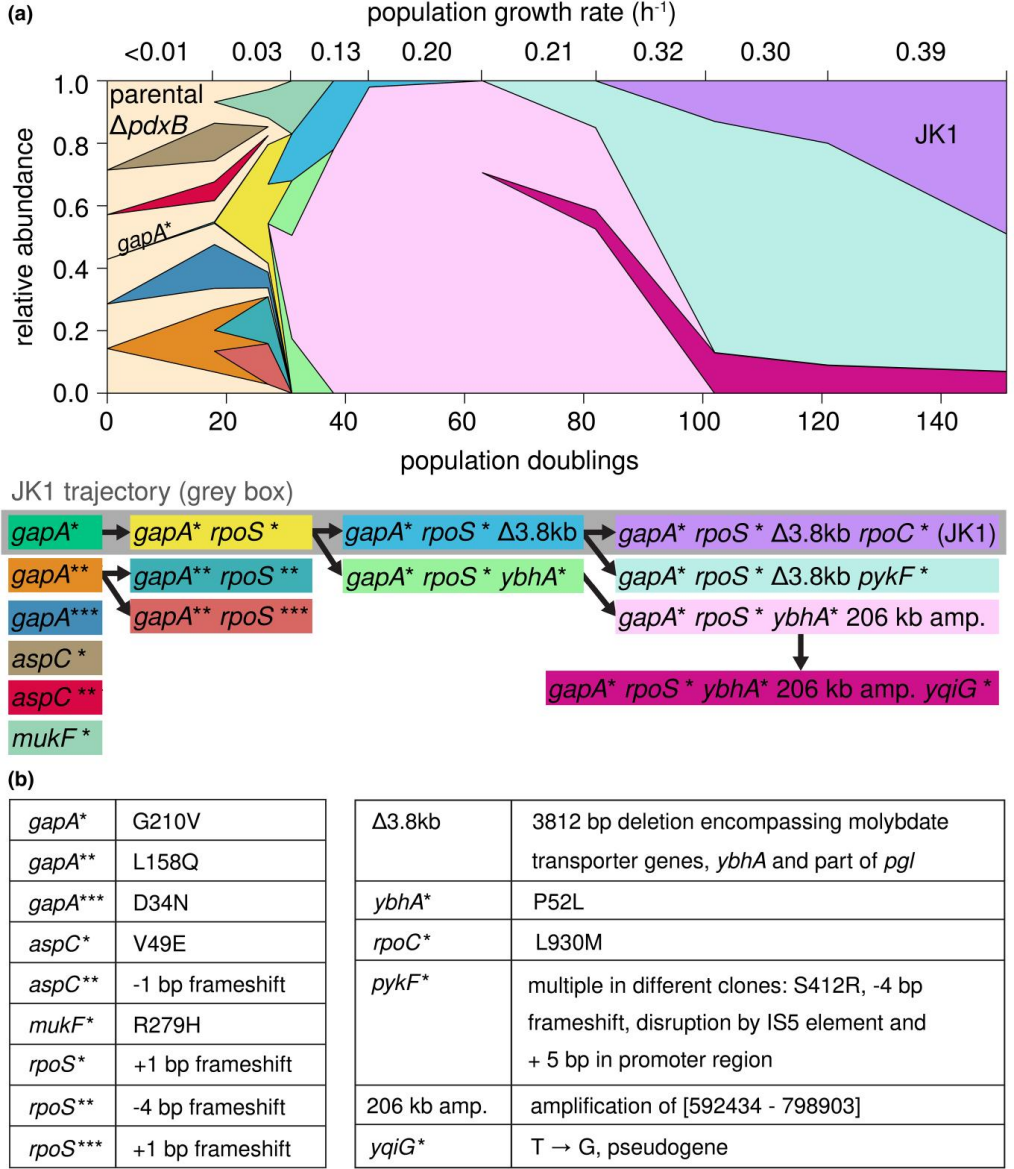
Complex population dynamics occurred during evolution of strain JK1. a) Muller plot tracking the abundance of clones throughout the evolution experiment. Population gDNA was sequenced after generations 18, 27, 31, 38, 44, 63, 82, 102, 121, and 151. Population growth rates at the time samples were taken for sequencing were estimated based on the initial and final values of OD_600_ for the previous passage. To simplify the analysis, the plot tracks only mutations that were found at two or more consecutive timepoints, were present in over 10% of the reads, or arose in a gene in which other mutations had already been found. b) Mutations listed in (a). Additional details are provided in supplementary Data Set S1, Supplementary Material online.

We sequenced population gDNA from samples archived during the evolution of JK1 to identify when mutations in dominant lineages occurred. Figure 2 summarizes the rise and fall of abundant clones over the course of 151 population doublings. Clones with different point mutations in *gapA* arose early (Fig. 2). All three mutations would be expected to diminish but not abolish the activity of GAPDH, which is essential for growth on glucose. We previously found that the *k*_cat_/*K_M_* of G210V GAPDH is 8-fold lower than that of the wild-type enzyme. L158Q GAPDH has normal catalytic activity but is less stable than the wild-type enzyme; its *T*_M_ is decreased from 72.6 ± 0.2 to 66.5 ± 0.4 °C. These mutations decrease GAPDH activity in lysates by 60% to 80% (Kim et al. 2019). A third mutation in *gapA* changes Asp34 to Asn. Asp34 coordinates the hydroxyl groups of the NAD(H) cofactor (PDB 1GAD); changing this residue to Asn should impair NAD(H) binding and catalytic activity.

Clones with mutations in *gapA* quickly acquired different frameshift mutations in *rpoS*. One *rpoS* mutation arose in the gapA* background in the lineage that eventually led to JK1. Two other *rpoS* mutations arose in the gapA** background. By 25 population doublings, strains with mutations in *gapA* and *rpoS* comprised 68% of the population. Clearly, this combination provides a significant improvement in fitness.

Two clones emerged from the gapA* rpoS* clone. The clone that led to JK1 (cyan in Fig. 2) had acquired a large deletion encompassing the molybdate transport operon, *ybhA*, and part of *pgl.* (This deletion will subsequently be referred to as Δ3.8 kb for simplicity.) Another clone (light green in Fig. 2) had acquired a point mutation in *ybhA* and then amplified a 206 kb region encompassing the mutant gene (ybhA*) (supplementary fig. S1, Supplementary Material online). This clone (pink in Fig. 2) nearly overtook the population within about 10 population doublings. The clone leading to JK1 (cyan in Fig. 2) was not detectable at 63 population doublings at 92× sequence coverage. However, additional mutations in either *rpoC* or *pykF* enabled the gapA* rpoS* Δ3.8 kb clone to overcome the previously dominant clone (pink in Fig. 2), possibly due to the metabolic burden imposed by the amplified 206 kb region.

### Impaired PLP Synthesis Compromises the Integrity of the Cell Wall

**Fig. 3.**
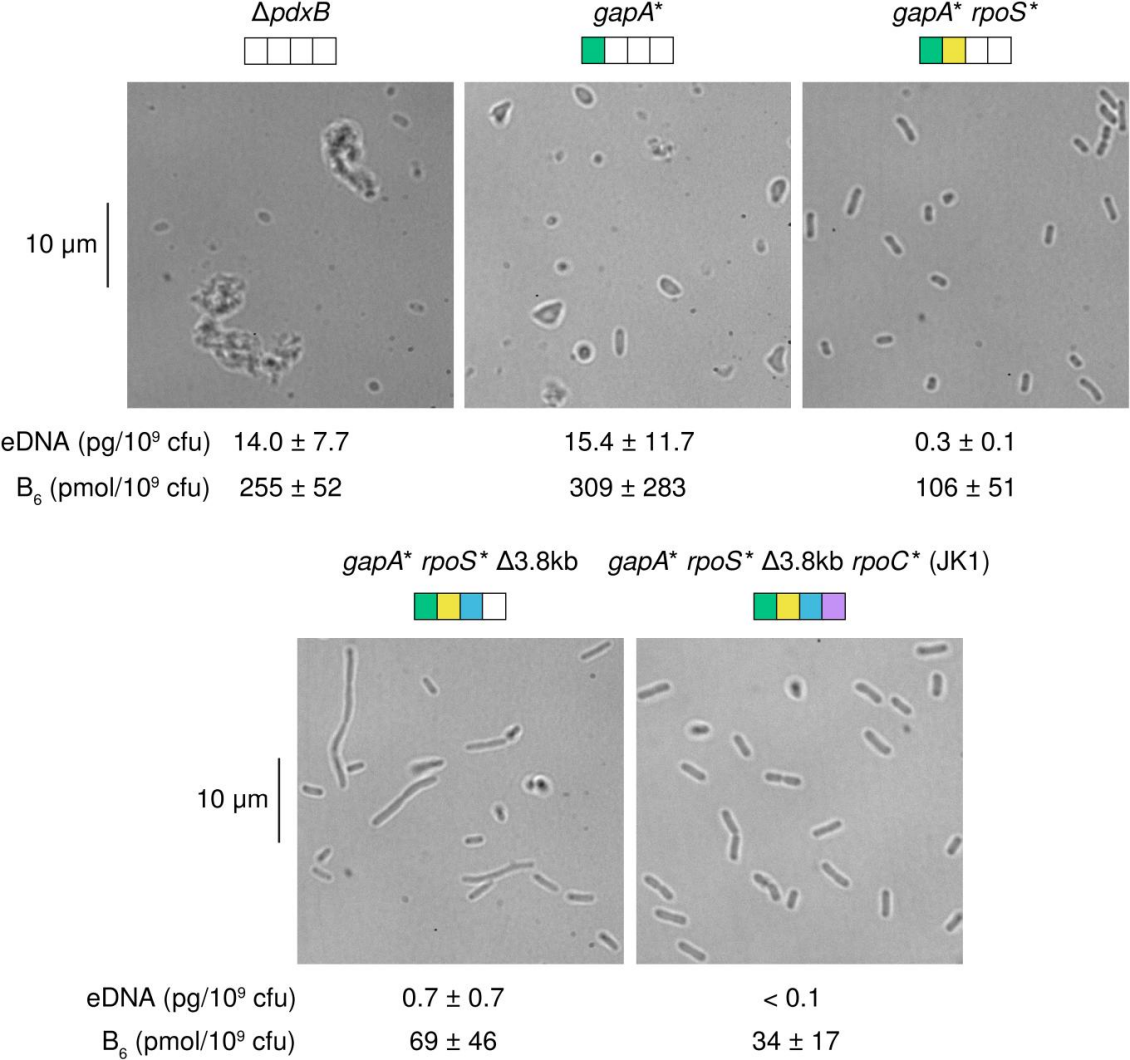
The parental Δ*pdxB E. coli* and intermediate strains along the trajectory toward JK1 exhibit odd cell morphologies. JK1 exhibits normal rod-shaped morphology and releases very little DNA into the medium from lysed cells. Amounts of extracellular DNA (eDNA) and B_6_ vitamers in the spent medium (indicative of cell lysis) are provided below each micrograph.

Synthesis of peptidoglycan requires substantial quantities of L-alanine, D-alanine, D-glutamate, and meso-diaminopimelic acid. Three of these compounds are produced by PLP-dependent enzymes. Not surprisingly, the Δ*pdxB* and gapA* strains show signs of a weak cell wall. These strains have abnormal morphologies (Fig. 3). Further, elevated levels of DNA and B_6_ vitamers (including PLP as well as other vitamers that can be converted to PLP, the active form of B_6_) are found in spent medium after growth of these strains. Impaired PLP synthesis apparently weakens the cell walls of these strains, leading to frequent lysis with release of cytoplasmic contents into the medium. Oddly, the gapA* rpoS* Δ3.8 kb strain was less prone to lysis but formed long filaments, suggesting a perturbed balance between cell elongation and cell division. In contrast, the evolved JK1 cells have normal morphology and release much less DNA and vitamin B_6_ into the spent medium than the parental Δ*pdxB* and the gapA* strains.

### Most Evolutionary Trajectories Toward JK1 Are Accessible

**Fig. 4.**
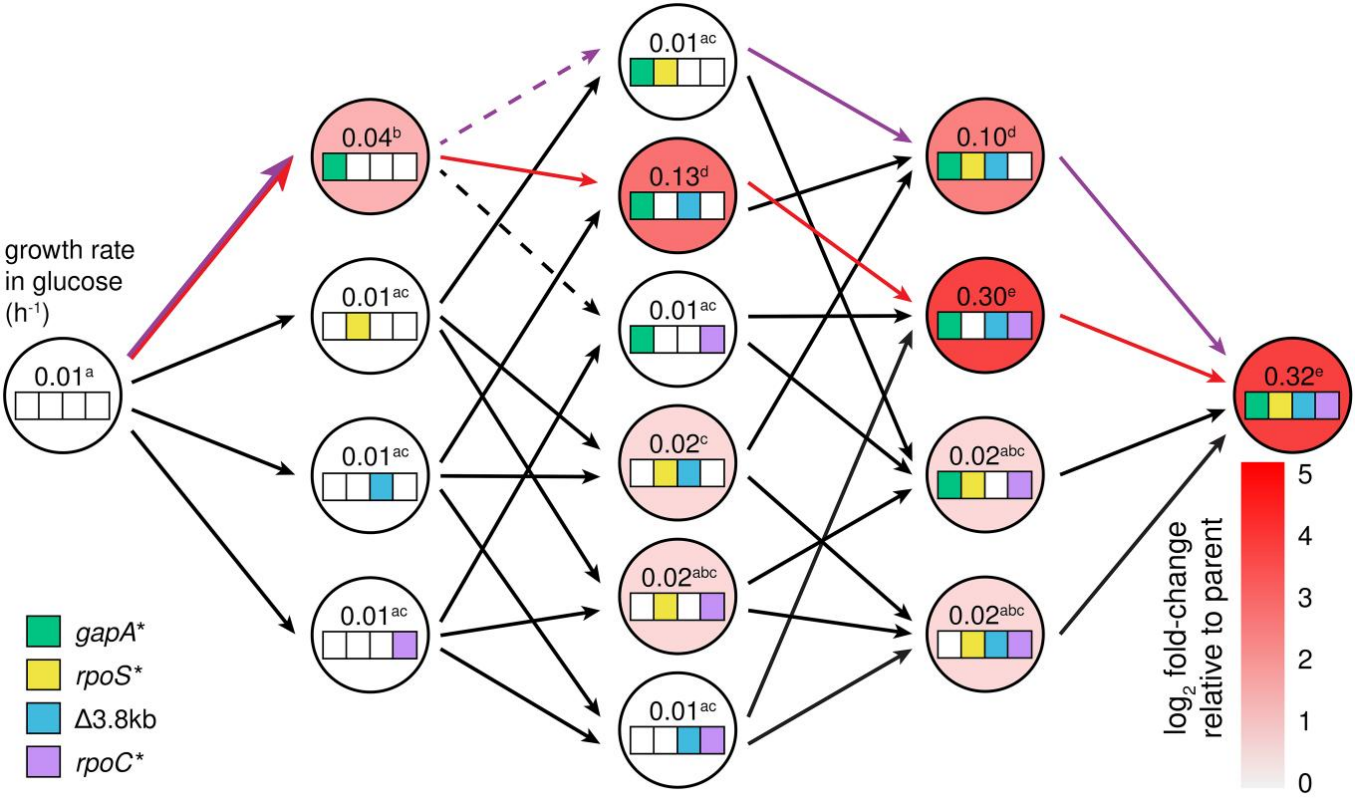
Fitness landscape showing the effect of each mutation on growth in M9/glucose. Superscripts represent statistical groups with *P*-adj < 0.05 after Dunnett T3 correction for multiple comparisons (GraphPad Prism 8). Purple arrows, the actual trajectory by which JK1 evolved; red arrows, the optimal trajectory. Dashed lines indicate steps that result in a statistically significant decrease in growth rate in M9/glucose.

Strain JK1 had accumulated four mutations in the order depicted in Fig. 2. To determine whether other evolutionary trajectories were accessible, we constructed all possible intermediates between Δ*pdxB E. coli* and JK1 and measured their growth rates in M9/glucose (Fig. 4; supplementary table S1, Supplementary Material online). The resulting fitness landscape reveals several interesting features. First, the gapA* mutation was the only one of the four mutations that increased the growth rate of the parental Δ*pdxB* strain. Second, the rpoS* mutation was not beneficial in any background and, indeed, reduced the growth rate of the gapA* strain by 4-fold. This finding is unexpected, since the Muller diagram in Fig. 2 clearly shows that *rpoS* mutations were advantageous in the background of a *gapA* mutation. We will return to this puzzling finding below. Third, the Δ3.8 kb mutation only improved growth rate when the *gapA* mutation was already present. Finally, the *rpoC* mutation was only beneficial when the gapA* and Δ3.8 kb mutations were already present.

The fitness landscape in M9/glucose (Fig. 4) reveals positive epistasis between the mutations that led to JK1. In the absence of epistasis, we would expect JK1 to grow 4-fold faster than Δ*pdxB E. coli*; the actual increase in growth rate is 32-fold. The rpoC*, rpoS*, and Δ3.8 kb mutations have little or no effect in most backgrounds, creating a smooth fitness landscape with 30 of 32 mutational steps accessible and all 16 intermediates accessible by at least one step. Only one trajectory (red arrows in Fig. 4) had significant increases in growth rate at each of the first three steps. Notably, the actual trajectory toward JK1 proceeded through one of only two mutational steps (dashed arrows in Fig. 4) that significantly reduced fitness.

### The gapA* Mutation Is the Critical First Step

The gapA* mutation is clearly the critical first step toward establishing the new protopathway. The gapA* mutation occurred first in the lineage leading to JK1. Two other clones also acquired mutations in *gapA* prior to other mutations. Clones that acquired mutations in other genes (*aspC* and *mukF*) were quickly outcompeted in the evolving population (Fig. 2).

**Fig. 5.**
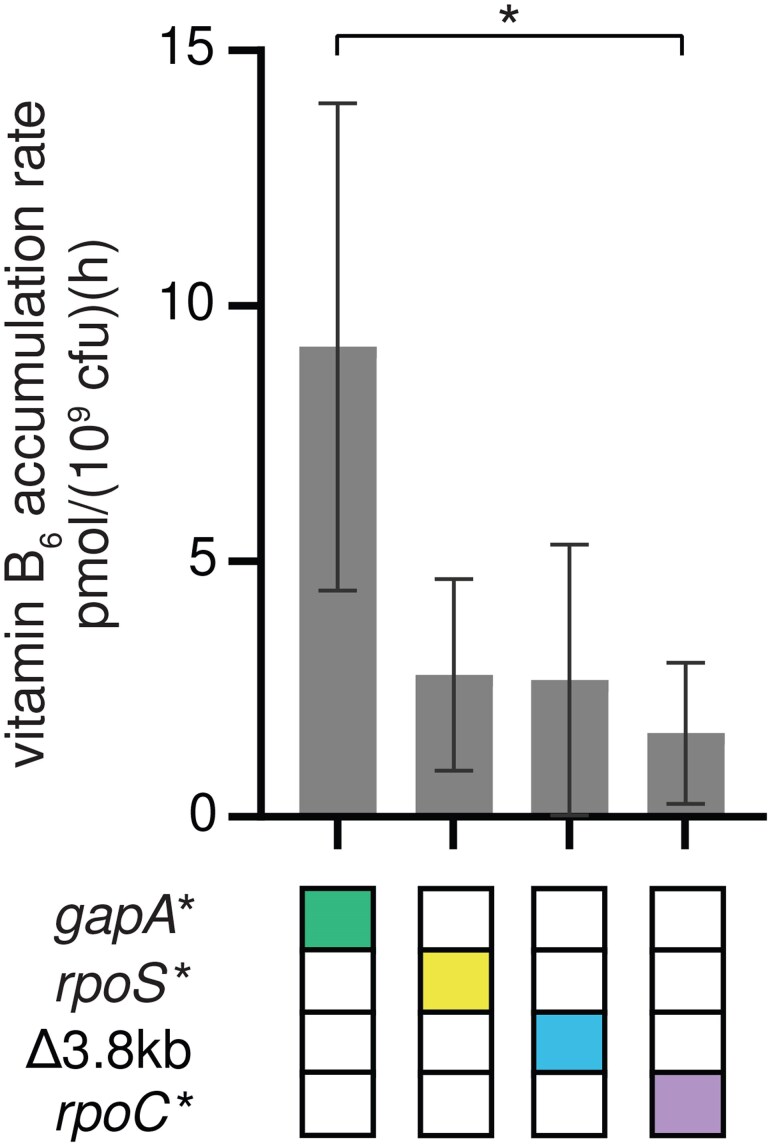
PLP accumulation rates in single mutants suggest that the gapA* mutation increases PLP synthesis. Error bars represent 95% confidence intervals. *P*-values adjusted by Dunnett T3 correction for multiple comparisons (GraphPad Prism 8). *P-adj < 0.05.

The gapA* mutation increased growth rate in M9/glucose by 4-fold in the parental background and up to 30-fold in other backgrounds (e.g. Δ3.8 kb rpoC* → Δ3.8 kb rpoC* gapA*) (Fig. 2; supplementary table S1, Supplementary Material online). To determine whether this increase in growth rate was due to an increase in PLP synthesis, we optimized a published procedure for measuring B_6_ vitamers (Thi Viet Do et al. 2012). We measured the total level of B_6_ vitamers in cell lysates and spent medium to calculate the B_6_ accumulation rate. (We term this an accumulation rate because B_6_ content is the net result of its synthesis rate minus its degradation rate.) Assuming that the total B_6_ content must be doubled each time the cells divide, we can calculate the rate of PLP accumulation by dividing the total B_6_ content per cfu by the doubling time. Obtaining accurate B_6_ levels for the parental strain was difficult because it was so fragile and prone to lysis (Fig. 3). However, the growth rates of the Δ3.8 kb, rpoS*, and rpoC* strains are similar to that of the parental strain, suggesting that the parental strain likely has a similar PLP accumulation rate, approximately 3 pmol/(10^9^ cfu)(h) (Fig. 5). The gapA* strain has a PLP accumulation rate of 9 pmol/(10^9^ cfu)(h) (Fig. 5), suggesting that the gapA* mutation increases PLP accumulation rate by about 3-fold, a value commensurate with the 4-fold increase in growth rate.

We previously showed that the gapA* mutation improves flux through the protopathway by alleviating feedback inhibition of SerA, the first enzyme in the serine synthesis pathway, by serine (Kim et al. 2019). The gapA* mutation reduces the activity of GapA in cell lysates by 80%. This bottleneck in glycolysis would be expected to decrease the concentration of downstream metabolites. One of these, 3-phosphoglycerate, is the native substrate of SerA. A reduction in the level of 3-phosphoglycerate should decrease production of serine, thereby reducing allosteric inhibition of SerA by serine. Previous metabolomics data showed that the four mutations in JK1 reduce the serine level to below the limit of detection (Kim et al. 2019). The improvement in PLP synthesis due to the gapA* mutation suggests that it plays a major role in reducing serine levels.

**Fig. 6.**
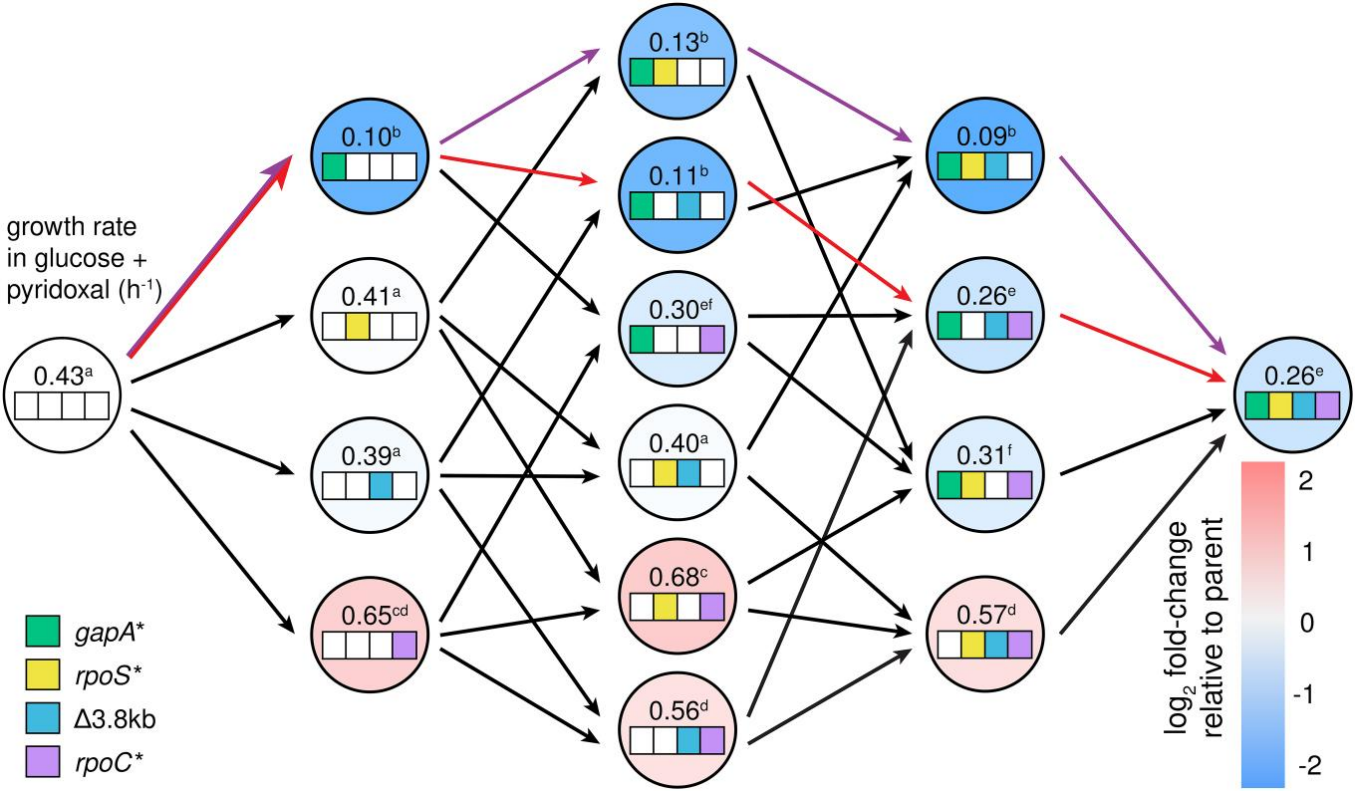
Fitness landscape showing the effect of each mutation on growth in M9/glucose + 10 µM pyridoxal. Superscripts represent statistical groups with *P*-adj < 0.05 after Dunnett T3 correction for multiple comparisons (GraphPad Prism 8). Purple arrows, the actual trajectory by which JK1 evolved; red arrows, the optimal trajectory.

Although the gapA* mutation is critical for increasing flux through the protopathway, it comes with a clear cost. Adding the gapA* mutation to every background decreases growth rate in nonselective conditions (M9/glucose plus pyridoxal, which can be converted to PLP by a salvage pathway) (Fig. 6; supplementary table S1, Supplementary Material online). This finding is not surprising because restricting flux through glycolysis should impair energy production and the biosynthetic processes required for cell division. Trade-offs caused by mutations that are beneficial under selective conditions but detrimental once selective pressures are relieved are common (MacLean et al. 2004; Rodriguez-Verdugo et al. 2014; Payen et al. 2016; Morgenthaler et al. 2019; Schmidlin et al. 2024). We refer to such mutations as “expedient” to denote their ability to quickly solve a problem under one condition, but at an overall fitness cost. The gapA* mutation is clearly expedient.

### The gapA* rpoS* Intermediate Strain in the Trajectory Toward JK1 Is a Cheater

Remarkably, JK1 evolved by a trajectory that passes through an intermediate whose growth rate in M9/glucose is slower than that of its progenitor; the addition of rpoS* to the gapA* background results in a significant decrease in growth rate in M9/glucose (Fig. 4; supplementary table S1, Supplementary Material online). Yet, the gapA* rpoS* clone outcompeted its progenitor, the gapA* clone (Fig. 2). Additionally, two other *rpoS* mutations arose in a clone with a different *gapA* mutation (teal and terracotta in Fig. 2). These results suggest that, counter to expectations based upon the fitness landscape in M9/glucose, loss-of-function mutations in *rpoS* were beneficial in the evolving population during the evolution of Δ*pdxB E. coli*.

Mutations in *rpoS* are common in experimental evolution in M9/glucose (Notley-McRobb et al. 2002; Maharjan et al. 2006; Ferenci 2008; Knoppel et al. 2018). If the rpoS* mutation were providing a general growth benefit, we would expect it to increase growth rate when selection for improved PLP synthesis is alleviated by the addition of pyridoxal, which can be converted to PLP via a salvage pathway, to the medium. However, the rpoS* mutation did not increase growth rate in M9/glucose + pyridoxal in any background (Fig. 6; supplementary table S1, Supplementary Material online). The detrimental effect of the rpoS* mutation when clones are grown in isolation suggests that the gapA* rpoS* strain is more fit in the context of the evolving population than it is on its own. To test this hypothesis, we measured the growth rates of the gapA* and gapA* rpoS* strains in spent medium collected after growth of the parental cells. The spent medium increased the growth rate of the gapA* rpoS* strain by 4.8-fold (supplementary fig. S2, Supplementary Material online), suggesting that it exploits resources released into the medium by lysis of the fragile parental Δ*pdxB* cells in the context of the evolving population.

RpoS serves as a global regulator by interacting with RNA polymerase to activate transcription of numerous genes important for redirecting cellular resources from growth toward survival (Hengge-Aronis 2002; Battesti et al. 2011). Consequently, RpoS expression is typically associated with a slowing of growth during stressful conditions or limited nutrient availability (Hengge-Aronis 2002; Notley-McRobb et al. 2002; Battesti et al. 2011; Bouillet et al. 2024). RpoS would be expected to be important for the gapA* strain that is handicapped by both poor PLP synthesis and diminished flux through glycolysis. Indeed, 39 genes in the RpoS regulon are upregulated in the gapA* strain compared with the parental Δ*pdxB* strain (supplementary fig. S3 and Data Set S2, Supplementary Material online). Several of the upregulated genes encode glycolytic enzymes, including glucose 6-phosphate isomerase, fructose bisphosphate aldolase class I and class II, GAPDH, and enolase. Activation of the stress response in the gapA* clone likely helps compensate for the decrease in glycolytic flux caused by the gapA* mutation. Consequently, loss of RpoS function is detrimental when the gapA* rpoS* clone is grown in isolation. However, loss of RpoS function in the gapA* rpoS* clone is clearly advantageous in the evolving population. We suspect that loss of RpoS allows this clone to avoid activating the programmed slowdown in growth orchestrated by RpoS, thereby giving it an advantage over the slower-growing parental strain and the three clones with mutations in *gapA*. Thus, this strain is a cheater, taking advantage of resources released by the lysis of fragile cells in the population but providing nothing in return. This notion is consistent with a recent report that loss-of-function mutations in *P. putida rpoS* allowed strains to cheat off other strains with intact *rpoS* (Robinson et al. 2020). The cheater is a viable evolutionary intermediate in the evolution of JK1 because the mutation that enabled cheating allowed the clone to persist long enough to acquire an additional beneficial mutation (see the following section). Interestingly, every successful clone by the end of the experiment originated from the cheater.

### The Δ3.8 kb Mutation Improves PLP Accumulation Rate in Backgrounds Containing the gapA* Mutation

**Fig. 7.**
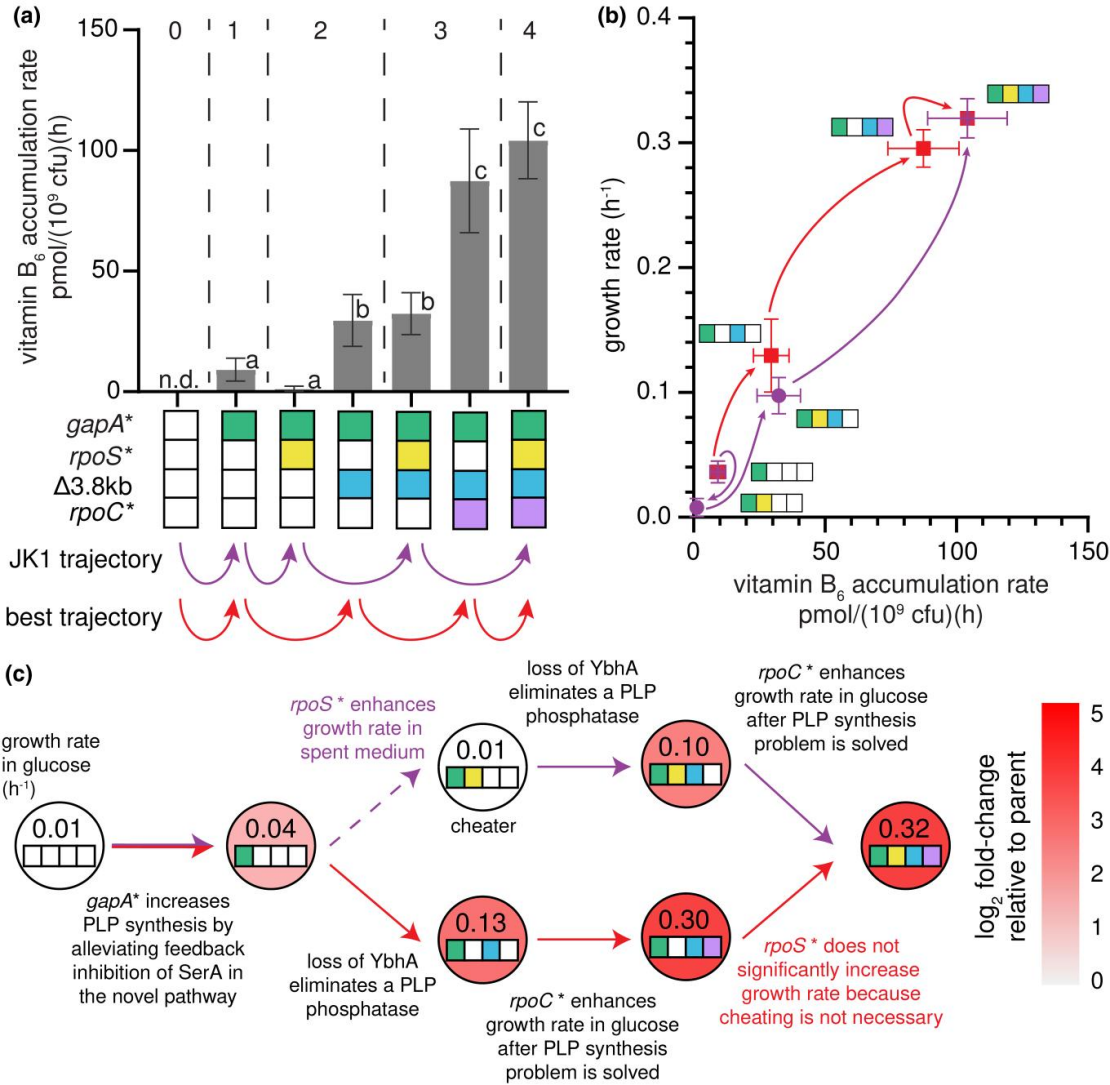
The combination of the gapA* and Δ3.8 kb mutations significantly increases the PLP accumulation rate. a) Vitamin B_6_ accumulation rates in the actual and best trajectories. Gray dashed lines separate the parent, single, double, triple, and quadruple mutant strains. Error bars represent 95% confidence intervals. Letters indicate statistical groups with *P*-adj < 0.05 after Dunnett T3 correction for multiple comparisons (GraphPad Prism 8). n.d., not done. b) Growth rate versus vitamin B_6_ accumulation rate. Arrows indicate evolutionary trajectories. Error bars represent 95% confidence intervals. c) Summary of the mutations and their effects in the trajectory leading to JK1 and the best trajectory.

The addition of the Δ3.8 kb mutation to the gapA* rpoS* clone causes a 10-fold increase in growth rate (Fig. 4; supplementary table S1, Supplementary material online) and a 32-fold increase in PLP accumulation rate (Fig. 7). The Δ3.8 kb mutation also increases growth rate in other gapA*-containing backgrounds, ranging from 3.3-fold (e.g. gapA* → gapA* Δ3.8 kb) to 30-fold (e.g. gapA* rpoC* → gapA* rpoC* Δ3.8 kb) (Fig. 4; supplementary table S1, Supplementary material online). The addition of the Δ3.8 kb mutation to backgrounds lacking the gapA* mutation results in at most a 2-fold increase in growth rate, suggesting that gapA* is required to unlock the full benefit of the Δ3.8 kb mutation.

The Δ3.8 kb mutation removes two genes of interest, *ybhA* and *pgl*. Loss of YbhA, a PLP phosphatase (Kuznetsova et al. 2006; Sugimoto et al. 2017), should spare PLP from degradation but would be of little help unless a prior mutation had increased flux through the protopathway. Parental Δ*pdxB* cells and others lacking the gapA* mutation likely have extremely low levels of free PLP, so YbhA would have little substrate in these cells. However, after the gapA* mutation increases flux through the protopathway, levels of PLP are apparently high enough for loss of YbhA to be beneficial. In addition to sparing PLP from degradation, the increase in PLP level resulting from the deletion of *ybhA* could increase flux through the protopathway. SerC, which catalyzes the transamination of DHOB in the protopathway (Fig. 1), requires PLP. Thus, an increase in PLP might increase the concentration of active SerC, further enhancing production of PLP.

We previously hypothesized that removal of *pgl* would result in diversion of GAP from glycolysis into the pentose phosphate pathway, thereby decreasing flux into lower glycolysis and contributing to the decrease in serine concentration that relieves feedback inhibition of SerA (Kim et al. 2019). However, a clone of gapA* rpoS* that acquired a point mutation in *ybhA* (with no effect on *pgl*) significantly increased in abundance in the population (Fig. 2), suggesting that the *ybhA* mutation alone is sufficient to cause a significant increase in growth rate.

The gapA* rpoS* Δ3.8 kb clone is the first intermediate in the trajectory to JK1 that has similar growth rates in M9/glucose (Fig. 4, µ = 0.10 h^−1^) and M9/glucose plus pyridoxal (Fig. 6, µ = 0.09 h^−1^), suggesting that its growth is not limited by PLP synthesis. However, the physiology of this strain is still clearly abnormal because the cells form long filaments (Fig. 3).

### The rpoC* Mutation Substantially Improves Growth Rate in Glucose

The addition of rpoC* to the gapA* rpoS* Δ3.8 kb strain results in a 3-fold increase in growth rate in M9/glucose (Fig. 4; supplementary table S1, Supplementary Material online) and a similar increase in M9/glucose plus pyridoxal. rpoC* increases growth rate in M9/glucose in one other strain, gapA* Δ3.8 kb, whose growth also does not appear to be limited by PLP accumulation (i.e. growth rate is similar in the presence and absence of pyridoxal). Thus, rpoC* confers an increase in growth rate only after PLP accumulation has been restored, suggesting that it simply improves growth rate in glucose. In agreement with this finding, the addition of rpoC* to almost every background improves growth rate in M9/glucose plus pyridoxal (Fig. 6; supplementary table S1, Supplementary Material online). Additionally, mutations in *rpoC* are frequently observed after adaptive laboratory evolution in glucose and glycerol and have many effects that improve growth rate in minimal medium (Conrad et al. 2010; Sandberg et al. 2014; Wytock et al. 2018).

Interestingly, the rate of PLP accumulation increases after the rpoC* mutation, even though the growth rate of the progenitor strain (gapA* rpoS* Δ3.8 kb) is no longer limited by PLP synthesis. Apparently, flux through the protopathway can be increased to keep up with the demand required by the faster growth conferred by the rpoC* mutation. However, we cannot rule out the possibility that some of the transcriptional changes caused by the rpoC* mutation play a role in increasing PLP synthesis. *rpoC* mutations are known to increase metabolic efficiency and anabolism during growth in minimal media and have pleiotropic effects on the transcriptome (Conrad et al. 2010), proteome, and metabolome (Cheng et al. 2014). For example, a mutation in *rpoC* perturbs the levels of 68 metabolites and 118 proteins when *E. coli* MG1655 is grown in glycerol as a sole carbon source (Cheng et al. 2014). Thus, it is difficult to determine whether the rpoC* mutation directly increased the PLP accumulation rate in JK1. Nevertheless, the rpoC* mutation restores normal cellular morphology (Fig. 3), possibly because the increased rate of PLP accumulation better supports peptidoglycan synthesis.

## Conclusion

By characterizing the fitness landscape for the evolution of JK1, we have identified how mutations led to the emergence of a novel protopathway (Fig. 7). The gapA* mutation increases the rate of PLP synthesis by reducing feedback inhibition of SerA, allowing it to better perform its new function in the protopathway. The next mutation, rpoS*, allows the gapA* rpoS* strain to cheat off other strains in the population. Since the stress response activated by RpoS shifts metabolism toward slow growth when the cells perceive stress or starvation (Notley-McRobb et al. 2002; Bouillet et al. 2024), loss of RpoS apparently allows the gapA* rpoS* strain to grow more rapidly using the abundant glucose in the medium as well as the metabolites released from lysis of the fragile parental Δ*pdxB* cells. The Δ3.8 kb mutation is only beneficial in backgrounds containing the gapA* mutation, suggesting that loss of the PLP phosphatase YbhA is only beneficial after PLP synthesis has been improved by the gapA* mutation. Finally, the rpoC* mutation increases the growth rate in glucose after the PLP accumulation rate no longer limits the growth rate. Interestingly, this final mutation provides an additional 3.3-fold bump in PLP accumulation rate by an unknown mechanism.

The “best” trajectory toward JK1 is the one with the largest fitness increase at each step. This trajectory (Fig. 7) also begins with the gapA* mutation but does not pass through the gapA* rpoS* cheater strain. Bypassing the cheater mutation enables the clone following the best trajectory to reach a growth rate comparable to that of JK1 in M9/glucose by only three mutations. If JK1 had been characterized solely by reverting individual mutations, rpoS* might have been mistakenly identified as a hitchhiker mutation (i.e. a mutation that confers no growth benefit but became fixed during evolution because it coincided with a beneficial mutation). However, its evolutionary significance is evident, as the gapA* rpoS* clone became a dominant clone in the population (Fig. 2) by exploiting the nutrients released from the fragile progenitor strains.

All clones in the population by the end of the evolution depicted in Fig. 2 arose from the gapA* rpoS* cheater strain; JK1 was the dominant clone. Several other successful clones (grouped together in turquoise in Fig. 2) also arose in the gapA* rpoS* Δ3.8 kb background by acquiring mutations in *pykF.* Mutations in *pykF* were also found in two other evolved populations of Δ*pdxB E. coli* (Kim et al. 2019). Mutations in *pykF* have been identified after long-term evolution of *E. coli* in glucose (Woods et al. 2006). Thus, the occurrence of multiple *pykF* mutations and the rpoC* mutation in the gapA* rpoS* Δ3.8 kb background is consistent with the conclusion that PLP accumulation rate is adequate at this stage and selection has shifted to favor mutants that grow faster in minimal medium containing glucose as a sole carbon source.

It is intriguing that the protopathway that emerged in JK1 differs from the previously identified underground pathway (Fig. 1). Elevation of flux through the underground pathway was achieved by overexpression of either NudL or ThrB on a pCA24N vector with a copy number of 300 to 400 (Soo et al. 2011) under control of the strong IPTG-inducible T5-lac promoter. Perhaps promoter mutations cannot produce enough of these enzymes to result in significant flux through the underground pathway depicted in Fig. 1. Interestingly, however, the endogenous level of ThrB appears to be sufficient when it is supplied with an elevated level of 4-hydroxythreonine, either via the underground pathway when NudL is overexpressed or via the protopathway when the gapA* mutation increases the activity of SerA.

Although the mutations in JK1 greatly increase growth rate in M9/glucose compared with the parental Δ*pdxB* strain, JK1 grows at only 50% of the rate of wild-type *E. coli* in M9/glucose (Kim et al. 2019). Previous work showed that the concentrations of 3-phosphoglycerate, a glycolytic intermediate, and serine, which is synthesized from 3-phosphoglycerate, are very low in JK1 (Kim et al. 2019). Thus, the mutations that improved PLP accumulation rate in JK1 caused significant perturbations to the metabolic network. In theory, the need for the damaging gapA* mutation could be alleviated by duplication and divergence of *serA* to evolve an efficient erythronate dehydrogenase that is not subject to inhibition by serine.

This work has provided an unprecedented look at the events that allow a bacterium to synthesize a newly needed metabolite, in this case, 4-phosphohydroxy-L-threonine. The protopathway that emerged in JK1 (Fig. 1) likely follows the course of a previously invisible underground pathway. Remarkably, just two mutations, a point mutation in *gapA* and loss of *ybhA*, appear to enhance PLP synthesis and decrease PLP degradation, respectively, to the point that growth rate is no longer limited by PLP levels. However, this protopathway is not an elegant solution to the problem of restoring PLP synthesis because it wastes ATP; a phosphate is removed in the first step of the protopathway and restored in the last step. Additionally, the mutations that enabled this pathway disrupted normal metabolism, leading to significant growth impairment. In theory, GAPDH and YbhA function could be restored after gene duplication and divergence led to a specialized erythronate dehydrogenase that is no longer subject to feedback inhibition by serine, but the wasting of ATP would still be a problem. The reservoir of promiscuous activities in the *E. coli* proteome might support additional underground pathways that are energetically more efficient, but for which elevation of flux via mutations is more difficult. For example, the promiscuous enzymes in an alternative underground pathway might be particularly inefficient, requiring multiple point mutations to improve catalytic efficiency, or the expedient mutations required to elevate flux might be too damaging. Current efforts in the lab are directed at evolving additional protopathways that might be better solutions to the problem of 4-phosphohydroxy-L-threonine synthesis.

The rapid emergence of a functional but inelegant protopathway for the synthesis of 4-phosphohydroxy-L-threonine has important implications for our understanding of the emergence of novel metabolic pathways. Selective pressure for the evolution of novel metabolic pathways has occurred countless times over the history of life on earth as organisms were exposed to new sources of carbon, nitrogen, and phosphate and attempted to produce complex natural products to better control their physical environments as well as competing and cooperating organisms. Our results raise the intriguing possibility that some of the pathways we see in extant organisms may not have been the first to emerge. Initial “quick-and-dirty” solutions may have provided organisms with a selective advantage when a new metabolic pathway was needed, giving them time to explore more propitious solutions. Alternatively, the massive collections of promiscuous enzymes in different organisms may have resulted in different protopathways, the most successful of which spread to other organisms by horizontal gene transfer. The window into early pathway evolution afforded by this study suggests that the process of evolving a novel metabolic is more complex and prone to missteps than previously appreciated.

## Materials and Methods

### Biological Resources

Strains, plasmids, and primers used in this work are included in supplementary tables S2 to S4, Supplementary Material online, respectively. Oligonucleotides and gene blocks (gBlocks) used to construct guide and donor plasmids are included in supplementary tables S5 and S6, Supplementary Material online, respectively. gBlocks used to construct protein expression plasmids are included in supplementary table S7, Supplementary Material online. Fragments used to construct donor and guide plasmids for genome editing by Gibson assembly are summarized in supplementary table S8, Supplementary Material online.

### Reagents

Chemicals (antibiotics, IPTG, etc.) and media (LB and SOC) were purchased from Sigma-Aldrich and Thermo-Fischer. Enzymes used for plasmid construction were purchased from NEB. Primers were obtained from IDT and Eurofins Genomics. gBlocks were obtained from IDT and Twist Bioscience.

### Statistical Analyses

Unless otherwise noted, statistical analyses were performed in GraphPad Prism 8. Statistical groups were assigned in R using the multcompLetters function.

### Population gDNA Sequencing

Aliquots (1 mL) of cultures archived during the evolution of JK1 had been maintained at −70 °C in M9/glucose containing 25% glycerol. Cells were harvested by scooping 400 to 600 µL of frozen culture into a 1.5 mL tube, thawing and centrifuging at 21,300*×g* for 1 min at room temperature. gDNA was extracted with an NEB Monarch gDNA kit (T3010) and submitted to SeqCenter for Illumina short-read sequencing. Reads were cleaned with Fastp 0.23.2 (Chen et al. 2018) using settings to trim the last base on each read and to remove reads with a phred quality less than 15 or with more than 20% unqualified bases, aligned with Bowtie2 2.1.0 (Langmead and Salzberg 2012) and analyzed with Breseq 0.35.4 (Deatherage and Barrick 2014) in polymorphism (mixed population) mode with CP009273.1 as the reference genome (Grenier et al. 2014). Read coverage exceeded 74× except for the final timepoint. For this sample, we sequenced gDNA prepared after growth of the culture overnight in LB to improve quantitation of *pykF* mutations, achieving 158× coverage. While the growth in LB might have resulted in some skewing of the population composition, the levels of the dominant mutations were comparable between sequence datasets. (Sequencing data after growth in LB are highlighted in yellow in supplementary Data Set S1, Supplementary Material online.)

The relative abundances of mutations in the evolving population of Δ*pdxB E. coli* that led to strain JK1 were determined by Breseq. (Frequencies of the gapA* and ybhA* mutations were determined in IGV (Thorvaldsdottir et al. 2013) when their abundances were too low to be registered by Breseq.) To simplify the analysis of the most important clones, we focused on mutations that were found at two or more consecutive timepoints and were present in over 10% of the reads or arose in a gene in which other mutations had already been found (supplementary Data Set S1, Supplementary Material online). Lineages were inferred based on correlations between the relative abundances of mutations across multiple passages. The 206 kb amplification was identified based on the read coverage map generated by Breseq (supplementary fig. S1, Supplementary Material online). The Muller diagram was generated in R 4.4.0 with the MullerPlot package (Farahour et al. 2022).

### Growth Rate Measurements

Cultures were routinely grown in M9/glucose (0.4% w/v) in the absence or presence of 10 µM pyridoxal. To measure growth rates in M9/glucose plus 10 µM pyridoxal, frozen glycerol stocks of *E. coli* strains were streaked onto LB agar plates and grown overnight at 37 °C. Single colonies were inoculated into 5 mL LB and grown overnight at 37 °C with shaking. Aliquots (250 µL) were inoculated into 25 mL of M9/glucose containing 10 µM pyridoxal in 40 mL vials in a FynchBio turbidostat. The cultures were grown at 37 °C with stirring (setting 8) and aeration provided by bubbling filtered air into the medium (25 mL/min). Cultures were started at an OD_600_ between 0.01 and 0.1 and then maintained at an OD_600_ between 0.14 and 0.16. (The FynchBio turbidostat measures IR light scattering rather than OD_600_. Standard curves were prepared to correlate light scattering to OD_600_.) The OD_600_ was recorded for at least 24 h. Growth rates were determined using data from the turbidostat and a Python script (Related Manuscript File 1) that fits the OD_600_ for each dilution cycle (or each 1- to 2-h interval if no dilution event occurred) to a single exponential. The µ values from multiple cycles (>6) were used to calculate the median for µ for each vial. Data reported are based on µ values for 7 to 13 biological replicates.

To measure growth rates in M9/glucose, starter cultures were grown in M9/glucose plus 10 µM pyridoxal as described above. After 18 to 24 h, cells were harvested by centrifugation at 4,500*×g* for 5 min at room temperature. Cell pellets were suspended in 1 mL of room-temperature M9/glucose. The suspensions were transferred to 1.5 mL tubes and centrifuged at 6,000×g for 1 min at room temperature to remove medium containing pyridoxal. The cells were washed five times with M9/glucose and suspended in 500 µL of M9/glucose before inoculation into 25 mL of M9/glucose in 40 mL vials in a FynchBio turbidostat to give an initial OD_600_ ≥ 0.14. The cultures were maintained in mid-log phase in the turbidostat as described above. OD_600_ was recorded for at least 72 h. Growth rates were determined as described above. Slow-growing strains showed three distinct growth phases: (i) initial fast growth that we attribute to residual intracellular PLP; (ii) a decline in OD_600_ that we attribute to lysis of some cells; and (iii) consistent slow growth once the cells acclimate to conditions in which the only source of PLP is endogenous synthesis. We only included growth data from phase three when determining growth rates for these strains. Some biological replicates obtained mutations leading to faster growth; data from these replicates were excluded. Data reported are based on µ values for 3 to 12 biological replicates.

To measure growth rates in spent medium from the parental strain, four 25 mL cultures of the parental Δ*pdxB* strain and two 25 mL cultures of the gapA* and gapA* rpoS* strains in M9/glucose were started in 40 mL vials of a FynchBio turbidostat as described above. After 24 h, by which time the OD_600_ was <0.16, the replicate cultures were combined, and spent medium from the parental Δ*pdxB* strain was collected by filtering the culture through a 0.22 µm PES filter membrane. gapA* and gapA* rpoS* cells were harvested by centrifugation at 4,500*×g* for 5 min at room temperature. The pellets were suspended in 1 mL of room-temperature M9/glucose and transferred to a 1.5 mL tube. Half of the cell suspension was inoculated into 25 mL of spent medium and the other half was inoculated into 25 mL of M9/glucose. These cultures were grown in the turbidostat as described above using either spent medium or M9/glucose to dilute cultures when they reached an OD_600_ = 0.16. OD_600_ was recorded for at least 48 h and growth rates were determined as described above. Data reported are based on µ values for 3 to 4 biological replicates originating from separate colonies.

### RNA Sequencing

Aliquots (500 µL) of cells were withdrawn from the turbidostat after 48 h of growth in M9/glucose, added to 1 mL of bacterial protect reagent (Qiagen 76526), mixed by vortexing, and incubated at room temperature according to the manufacturer's protocol. The suspended cells were centrifuged for 10 min at 5,000×g at room temperature. The supernatants were discarded and the cell pellets were flash frozen and maintained at −70 °C. Subsequently, cell pellets were thawed at room temperature and RNA was extracted with an RNeasy Mini Kit (Qiagen 74104) according to the manufacturer's protocol. Purified RNA samples were submitted to SeqCenter for Illumina short-read sequencing. Reads were cleaned with Fastp 0.23.2 using settings to remove reads with phred quality less than 15 or with more than 20% unqualified bases and aligned to the *E. coli* CP009273.1 reference genome (Grenier et al. 2014) with Bowtie2 2.1.0. Bowtie alignment files were converted to sorted bam files with SAMtools (Danecek et al. 2021). Read counts for each gene were produced by HTSeq (Putri et al. 2022). Data analysis and visualization were performed with edgeR (Robinson et al. 2010). Data are provided in supplementary Data Set S2, Supplementary Material online.

### Microscopy

Slides for immobilization of bacteria were prepared by pipetting 10 µL of 0.01% poly-L-lysine hydrobromide solution (PLL, MP Biomedicals 150176) onto the center of a Premium Superfrost Plus glass slide (VWR 48311-601) and spreading the solution over a 1.5 × 1.5 cm square with a plastic cell spreader. Slides were dried at room temperature for 1 h. One milliliter of deionized H_2_O was used to wash the slide and remove excess PLL. Slides were dried and stored at room temperature for no more than 3 d.

Aliquots (200 µL) of cells were sampled from the turbidostat after a stable growth rate had been achieved and placed in the middle of a prepared slide. Cells were immobilized on the surface for 20 min. Unbound cells were removed by washing the slide with 1 mL M9/glucose. The cell-containing region was covered by a VWR glass coverslip No 1.5 (48366-227). Prepared slides were imaged within 1 h on a Yokogawa/Olympus CV1000.

### Quantification of Vitamin B_6_ in Cell Lysates and Spent Media

Total vitamin B_6_ content in cell lysates and spent media was determined by a modification of a literature procedure to enzymatically convert all B_6_ vitamers to 4-pyridoxolactone (4-PLA; supplementary fig. S4, Supplementary Material online), a highly fluorescent molecule, prior to quantitation by HPLC (Thi Viet Do et al. 2012). Cells were washed and grown as described above for determination of growth rates. Samples were harvested from the turbidostat after stable growth rates were achieved in M9/glucose. Three aliquots (7.5 mL) were withdrawn from each vial with sterile 10 mL syringes. Three smaller aliquots (100 µL) were also withdrawn and plated for enumeration of colony-forming units (CFUs) (described below). The samples were transferred to a cool (15 °C), dimly lit room for processing to minimize photodecomposition of B_6_ vitamers (Reiber 1972; Saidi and Warthesen 1983). Cultures were pushed through a 0.22 µm MCE 25 mm syringe filter (CellTreat CT-229750), and ∼1 mL of spent medium was collected in an opaque black tube. Cells were then collected by washing the filter in the reverse direction using 1 mL 10 mM CHES buffer, pH 9.0. The cell suspension was transferred to a tube containing 1,200 mg of 100 µm zirconium beads (Ops Diagnostics 10010026). An aliquot (50 µL) was removed and diluted in 450 µL of M9/glucose to quantify OD_600_ and CFUs. Spent media and cell suspensions were stored at −70 °C until processing for quantification of B_6_ vitamers.

Frozen cell suspensions with 1,200 mg of 100 µm zirconium beads (Ops Diagnostics 10010026) were thawed in a 15 °C water bath for 5 min. Cells were lysed by shaking at full speed for 20 min on a horizontal microtube holder attachment (SI-H524) mounted on a Vortexer 2.0. Lysates were centrifuged for 1 min at 6,000*×g* and the supernatants were filtered through a 0.22 µm MCE 13 mm syringe filter (CellTreat CT-229750). An aliquot (200 µL) of the filtered cell lysate was placed in an opaque black tube containing 20 µL of premixed solution comprised of His-tagged recombinant Proteinase K (Abcam ab281339), 2.5 U His-tagged Benz-Neburase (GenScript Z03626-100), 5.5 mM MgCl_2_, and 550 mM ammonium acetate, pH 9.0. The reaction mixture was incubated in a water bath for 2 h at 37 °C to digest nucleic acids and proteins and release covalently attached PLP from lysine residues. (In the absence of a stable enzyme active site, the linkage between PLP and lysine residues in digested peptides is hydrolyzed at pH 9.0.) The remaining lysate was used for DNA and protein quantitation (described below). After protease digestion, 5 µL of 45 mM phenylmethylsulphonyl fluoride (PMSF) dissolved in ethanol was added and the samples were tumbled for 10 min to inactivate proteases. An aliquot (165 µL) of 50 mM ammonium acetate, pH 9.0, containing 2 mM pyruvate, 2 mM oxidized nicotinamide adenine dinucleotide (NAD^+^), 5 µM flavin adenine dinucleotide (FAD), 0.5 mM MgCl_2_, 50 U recombinant shrimp alkaline phosphatase (rSAP, NEB M0371L), 0.14 µM pyridoxine oxidase (PNOX), and 0.22 µM pyridoxamine-pyruvate aminotransferase (PPAT) was added to the samples, followed by 60 µL of 0.53 µM pyridoxal 4-dehydrogenase (PLDH). The resulting mixture was incubated in a 37 °C water bath for 1 h to convert B_6_ vitamers to 4-PLA. Purification and assays of PLDH, PPAT, and PNOX are described below. His-tagged proteinase K (20 µL of 10 mg/mL) was added and the reaction mixture was incubated in a 37 °C water bath for 1 h to digest the conversion enzymes PNOX, PPAT, and PLDH. Ni-NTA resin (10 µL, Cube Biotech 74105) was added to bind the His-tagged proteinase K and the reaction mixture was tumbled for 10 min. Finally, the reaction was filtered through a 0.22 µm MCE 13 mm syringe filter (CellTreat CT-229750) into 400 µL glass inserts (Agilent 5181-3377), placed in HPLC vials, and capped with a septum cap.

Spent medium samples were prepared and B_6_ vitamers were converted to 4-PLA as follows. Frozen spent media samples were thawed in a 15 °C water bath for 5 min and an aliquot (195 µL) was placed in an opaque black tube. EDTA (22 µL of 100 mM, pH 9.0) was mixed with the spent medium, followed by 8 µL of 1 M NaOH. An aliquot (165 µL) of 100 mM ammonium acetate, pH 9.0, containing 2 mM pyruvate, 2 mM oxidized nicotinamide adenine dinucleotide (NAD^+^), 5 µM flavin adenine dinucleotide (FAD), 1 mM MgCl_2_, 50 U rSAP (NEB M0371L), 0.14 µM PNOX, and 0.22 µM PPAT was added to the tumbled samples, followed by 60 µL of 0.53 µM PLDH. The reaction was incubated in a 37 °C water bath for 2 h to convert B_6_ vitamers to 4-PLA. Conversion enzymes were digested with His-tagged proteinase K and the solution was filtered as described above.

4-PLA was quantified by HPLC. The HPLC system consisted of an Agilent 1100 degasser (G1379A), a quat pump (G1311A), an autosampler (G1367A) with a 1290 thermostat (G1330B), and a fluorescence detector (G1321A). An Infinitylab Poroshell 120EC-C18 column (4.6 × 250 mm, 2.7 µm, Agilent 690975-902) with an Infinitylab Poroshell 120EC-C18 guard column (4.6 × 5 mm, 2.7 µm, Agilent 820750-911) was used for the separation of compounds. All samples were maintained in the dark at 4 °C in the autosampler prior to injection on the column. A standard curve of 4-PLA (Sigma 02582) from 0 to 40 nM was run for each set of 16 samples. After injection of 40 µL samples, 4-PLA was eluted with two column volumes of mobile phase A (10 mM ammonium acetate, pH 8.75, containing 5% [v/v] acetonitrile) at a flow rate of 0.75 mL/min. The column was washed with five column volumes of mobile phase B (95% [v/v] acetonitrile/water) and re-equilibrated with three column volumes of mobile phase A. The fluorescence intensity of the eluate was monitored at 425 nm (excitation at 355 nm) with a gain of 13 and response time of 4 s. Peaks were automatically integrated by Chemstation.

### Enumeration of CFUs

Aliquots (100 µL) of diluted cultures were spread on LB agar plates and incubated at 37 °C for 12 to 16 h. Plates were imaged with a digital camera in an imaging box (Smith and Schuster 2021), and colonies were counted with a custom Matlab script (Related Manuscript File 2). Plates with fewer than 10 colonies and more than 2,000 colonies were excluded from CFU calculations. OD_600_ and CFU were linearly correlated (3.52 × 10^8^ CFU/OD_600_, *r*^2^ = 0.76) and did not change between cell types, allowing the number of CFUs to be calculated from OD_600_.

### Quantification of DNA in Lysates and Spent Medium

DNA in lysate and spent medium samples was quantified using the Qubit HS DNA Detection Kit (ThermoFisher Q33231) in a 96-well plate. A solution comprised of 0.5 µL Component A and 89.5 µL Component B was mixed with an aliquot (10 µL) of the sample or DNA standard (concentrations ranging from 0.25 to 6 µmol/mL for lysate samples or 0.0078 to 6 µmol/mL for spent medium samples) and emission at 532 nm (excitation at 502 nm) was measured in a Thermo Varioscan 3.01.15 plate reader. The fluorescence of the standards was fitted to a quadratic curve and the regression coefficients were used to calculate the DNA concentrations in the samples.

### Quantification of Protein in Lysates

Protein in lysates was quantified using a Coomassie protein assay in a 96-well plate. An aliquot (75 µL) of Coomassie protein assay reagent (Thermo 1856209) was mixed with 25 µL of the sample or BSA standard (BioRad 500-0207) (concentrations ranging from 2.5 to 40 µmol/mL) and absorbance at 595 nm was measured in a Thermo Varioscan 3.01.15 plate reader. The standards were fitted to a quadratic curve and regression coefficients were used to calculate the protein concentrations in the samples.

### Protein Purification

gBlocks encoding enzymes required for analysis of B_6_ vitamers were ordered from Twist Bioscience. The gBlock encoding PLDH was assembled into a pET28 vector with a His_10_-SUMO tag incorporated at the N-terminus (pKAW077, supplementary table S2, Supplementary Material online). The gBlocks encoding PNOX and PPAT were cloned into a pET46 vector with a His_6_ tag incorporated at the N-terminus (pKAW110 and pKAW111, supplementary table S2, Supplementary Material online). The expression plasmids were introduced into *E. coli* NiCo21(DE3) (supplementary table S1, Supplementary Material online) by electroporation.

Expression and purification of PLDH were carried out as follows. A pipette tip was used to inoculate a small quantity of a frozen culture of *E. coli* NiCo21(DE3) containing pKAW077 into 10 mL of LB containing 50 µg/mL kanamycin in a 50 mL flask. The cultures were grown at 37 °C overnight with shaking and then inoculated into 1 L of TB containing 50 µg/mL kanamycin. After reaching an OD_600_ of 0.5 to 0.6, the culture was cooled to room temperature and isopropyl β-D-1-thiogalactopyranoside (IPTG) was added to a final concentration of 1 mM to induce protein expression. The culture was maintained at 25 °C with shaking for 16 h and cells were harvested by centrifugation at 6,000*×g* for 20 min at 4 °C. The cell pellet was suspended in 35 mL cold purification buffer with protease inhibitor cocktail (Thermo Scientific A32955) and lysozyme (1 mg/mL) and incubated for 1 h at 4 °C with tumbling. PLDH purification buffer consisted of 20 mM potassium phosphate buffer, pH 8.0, containing 0.1% (v/v) 2-mercaptoethanol, 10% (v/v) glycerol, and 1 mM EDTA (Yokochi et al. 2006). Cells were lysed by sonication (5 cycles, 10 s on, 20 s off) on ice. The disrupted cells were centrifuged at 20,000*×g* at 4 °C for 20 min to remove cell debris. Protein was purified using a hand-packed Ni-NTA resin column (300 µL bed volume of Cube Biotech 74105) using a step gradient of PLDH purification buffer containing increasing amounts of imidazole (10 mL of 20 mM, 5 mL of 50 mM, 5 mL of 100 mM, 5 mL of 200 mM, and 5 mL of 500 mM). SDS-PAGE was used to identify fractions containing PLDH. Fractions from the 200 and 500 mM imidazole elutions were combined. PLDH was further purified by size exclusion chromatography (Superdex 200 10/300 GL, 24 mL bed volume) with PLDH storage buffer (20 mM sodium carbonate, pH 9.0, containing 0.1% (v/v) 2-mercaptoethanol, 10% (w/v) glycerol, and 1 mM EDTA) as a running buffer. The final protein concentration was calculated using the A_280_ and the PLDH extinction coefficient calculated with Expasy ProtParam (https://web.expasy.org/protparam/). Purified protein was flash frozen and stored at −70 °C.

Expression of PPAT and harvesting of cells were carried out as described above for PLDH. PPAT purification buffer A consisted of 20 mM sodium phosphate, pH 7.4, containing 300 mM NaCl. Cells were lysed by sonication (5 cycles, 10 s on, 20 s off) on ice and centrifuged at 20,000*×g* at 4 °C for 20 min to remove cell debris. Residual cell debris was removed by filtration through a 0.45 µm 33 mm PVDF syringe filter (Millipore SLHVR33RS). Proteins were purified with a 1 mL HisTrap FF (Cytiva 17531901) column on an AKTA FLPC using a 30 mL linear gradient of PPAT purification buffer A and buffer B (buffer A plus 500 mM imidazole). Protein elution was monitored by A_280_ and fractions containing PPAT were identified by SDS-PAGE. PPAT was further purified by size exclusion chromatography (Superdex 200 10/300 GL, 24 mL bed volume) with PPAT purification buffer A (20 mM sodium phosphate, pH 7.4, containing 300 mM NaCl) as a running buffer. Protein elution was monitored by A_280_ and fractions containing PPAT were identified by SDS-PAGE. The final protein concentration was calculated using the A_280_ and the extinction coefficient calculated with Expasy ProtParam. Purified protein was flash frozen and stored at −70 °C.

Expression of PNOX required the chaperones GroEL and GroES (Yuan et al. 2004), which were overexpressed from pGro7 (Takara Bio 3340). Purification of PNOX proceeded as described above with a few modifications. Overnight starter cultures of *E. coli* NiCo21(DE3) containing pKAW111 and pGro7 were grown in LB containing 50 µg/mL kanamycin to maintain the overexpression plasmid and 30 µg/mL chloramphenicol to maintain pGro7; expression cultures also contained 50 µg/mL kanamycin and 30 µg/mL chloramphenicol. The cell pellet was suspended in PNOX purification buffer (20 mM potassium phosphate buffer, pH 8.0, containing 300 mM NaCl, 5 µM FAD, 0.1% [v/v] 2-mercaptoethanol, 10% [w/v] glycerol, 1 mM EDTA, and 0.01% [v/v] Tween 20) as described previously (Yuan et al. 2004). Cells were lysed and separated from cell debris as described above. Protein was purified using a hand-packed benchtop Ni-NTA resin column (300 µL bed volume of Cube Biotech 74105) using a step gradient of PLDH purification buffer containing increasing amounts of imidazole (10 mL of 20 mM, 5 mL of 50 mM, 5 mL of 100 mM, 5 mL of 200 mM, and 5 mL of 500 mM). SDS-PAGE was used to identify fractions containing PLDH. Fractions from the 100 and 200 mM imidazole elutions were combined. Buffer exchange was performed by several steps of concentration by centrifugation at 6,000*×g* for 20 min at 4 °C with a 30 kDa-cutoff membrane centrifugal filter (Millipore UFC9030) and dilution in a PNOX storage buffer (50 mM Tris buffer, pH 8.0, containing 5 µM FAD, 0.1% [v/v] 2-mercaptoethanol, 10% [w/v] glycerol, 1 mM EDTA and 0.01% [v/v] Tween 20). Purified protein was flash frozen and maintained at −70 °C.

### Enzyme Assays

The activities of PLDH, PPAT, PNOX, and rSAP were measured under the conditions used to convert B_6_ vitamers to 4-PLA (50 mM ammonium acetate, pH 9.0, containing 0.5 mM MgCl_2_, 2.5 µM FAD, 1 mM NAD^+^, and 1 mM pyruvate). Reaction mixtures were incubated in wells of a 96-well flat-bottom plate in a Varioskan plate reader for 5 min while measuring the background change in A_340_ every min. Reactions were initiated by the addition of pyridoxal, pyridoxamine, pyridoxine, or PLP to a final concentration of 1 mM. This assay procedure was used to determine the amount of each enzyme required to convert >99% of the B_6_ vitamers to 4-PLA in 30 min. All enzymes were stable at −80 °C for at least 6 months.

### Genome Editing

A helper plasmid (pDY118A) encoding Cas9, I-SceI, and the λ Red genes (alpha, beta, and gam) was transformed into Δ*pdxB*::*kanR E. coli* BW25113 from the Keio collection (Baba et al. 2006) as described previously (Yang et al. 2024). The four singly edited strains were constructed from the Δ*pdxB*::*kanR* strain as described below. Strains with multiple edits were constructed by successively adding edits to the singly edited strains.

We generated donor and guide plasmids containing I-SceI recognition sites and a *sacB* counter-selection marker from previously constructed donor plasmids pDonor1 and pDonor2 (Yang et al. 2024), respectively. Donor plasmids contain both an editing cassette and an sgRNA sequence, while guide plasmids contain only an sgRNA sequence. The I-SceI recognition sites and *sacB* counter-selection marker were included to aid plasmid curing after genome editing. However, leaky expression of I-SceI from the pDY118A helper plasmid proved to be efficient enough to cure the donor and guide plasmids so that neither I-SceI induction nor SacB counter-selection was necessary. PCR amplicons, gBlocks, and annealed primers used for Gibson assembly of donor and guide plasmids for genome editing are listed in supplementary table S7, Supplementary Material online. All PCR reactions were carried out using the manufacturer's protocol for NEB Q5 High-Fidelity DNA polymerase M0492L. Amplicons from plasmid templates were purified using a Monarch PCR & DNA purification kit (T1130L) and the template plasmid was removed by restriction digestion with NEB DpnI (R0176L) following the manufacturer's protocol. Amplicons of the correct size were purified using a GeneJET Gel Extraction kit (K0691). Plasmids were assembled by Gibson Assembly. Fragments were mixed in 1:1 molar ratios with 50 to 100 ng of backbone DNA. Homemade Gibson Master mix was made according to the recipe available at dx.doi.org/10.17504/protocols.io.n9xdh7n using NEB Taq DNA Ligase (M0208L), NEB T5 Exonuclease (M0663L), and NEB Q5 Hi-Fi DNA Polymerase (M0491L). Five microliters of mixed fragments were added to 15 µL of Gibson Master Mix and incubated at 50 °C for 2 to 16 h. Reaction mixtures were placed on Millipore MCE 0.025 µm membranes (VSWP01300) and dialyzed against ultrapure H_2_O for 1 to 2 h to remove salts. Assembled plasmids were transformed into *E. coli* DH5a by electroporation. Correct plasmid construction was verified by whole plasmid sequencing before genome editing procedures.

Cas9-assisted lambda Red recombination was used to introduce the 3,812 bp deletion (Δ3.8 kb). A guide plasmid encoding an sgRNA targeting *ybhA* was assembled from the backbone of pDonor1-SacB, excluding the editing cassette, and a sgRNA sequence made by annealing two complementary 60-nt primers (supplementary tables S4 and S7, Supplementary Material online), resulting in a 20 bp ds sgRNA sequence flanked by 20 bp extensions for Gibson assembly. Editing was performed as described previously (Yang et al. 2024) except that 30 µg/mL rather than 34 µg/mL chloramphenicol was used to maintain pDY118A. Editing was confirmed by colony PCR and Sanger sequencing.

Two-step Cas9-assisted lambda Red recombination was used to introduce the gapA* and rpoC* mutations. Because these genes are essential, we introduced multiple synonymous mutations into the genes in the first step of editing to maintain the essential function. In the second step, the region containing the multiple synonymous mutations was targeted by an sgRNA to enable replacement with the edited sequence provided as a linear editing cassette (rather than as a cassette in a donor plasmid). Round 1 donor plasmids (supplementary fig. S5, Supplementary Material online) used to introduce multiple synonymous mutations into the genome within 20 bp of the intended edits were constructed by Gibson assembly of the backbone of pDonor2-SacB with a gBlock (IDT) containing the synonymously recoded gene segment and extensions required for Gibson assembly (supplementary table S7, Supplementary Material online). In the second step, a guide plasmid was used to target the synonymously recoded site in the genome. Guide plasmids (supplementary fig. S6, Supplementary Material online) were constructed by Gibson assembly of a backbone amplified from pDonor1-SacB (excluding the previous sgRNA sequence) with an sgRNA sequence made by annealing two complementary oligonucleotides (supplementary table S4, Supplementary Material online). Linear editing cassettes were amplified from JK1 gDNA using primers KW019 and KW020 for gapA* and KW013 and KW014 for rpoC* (supplementary table S3, Supplementary Material online). Editing was performed as described previously (Yang et al. 2024) except that 100 µg/mL streptomycin rather than 100 µg/mL ampicillin was used for selection of guide plasmids.

I-SceI-assisted editing (Kim et al. 2014) was used to introduce the rpoS* mutation. Edits were introduced using a three-part dsDNA editing cassette comprised of: first, a segment containing a homology arm (HA) upstream of the edit, the edit, and a short downstream HA; second, a segment containing an 18-bp I-SceI cut site and a streptomycin resistance gene; and third, a final segment containing a short HA upstream of the edit, the edit, and a downstream HA. The editing cassette was amplified from pKAW066 using the manufacturer's protocol for NEB Q5 Hot Start DNA Polymerase (M0494L) and primers KW059 and KW385 (supplementary table S4, Supplementary Material online). Lambda Red proteins were expressed and cells were prepared for electroporation as described previously (Datta et al. 2006). The editing cassette (100 ng) was introduced into washed competent cells (50 µL) by electroporation, after which the cells were added to 1 mL SOC at 30 °C and allowed to recover for 3 h and then plated on LB agar plates containing 30 μg/mL chloramphenicol to maintain pDY118A and 50 μg/mL streptomycin to select for cells that had successfully recombined the editing cassette into the genome. After overnight incubation at 30 °C, colonies were streaked onto LB agar plates containing 30 μg/mL chloramphenicol and 50 μg/mL streptomycin and grown overnight at 30 °C to ensure elimination of unedited cells. Individual colonies were then streaked onto fresh LB agar plates containing 30 μg/mL chloramphenicol and 1 mM IPTG to induce I-SceI expression and promote RecA-mediated recombination to obtain the scarless edit. After overnight incubation at 30 °C, colonies were screened for incorporation of the desired edit.

Plasmids were cured from edited colonies by overnight growth in 5 mL LB at 37 °C with shaking, followed by streaking onto LB agar plates. After overnight growth on LB agar plates at 37 °C, individual colonies were streaked onto LB plates containing: (i) no antibiotics; (ii) 30 µg/mL chloramphenicol to test for retention of the helper plasmid pDY118A; and (iii) 100 µg/mL ampicillin or streptomycin to test for retention of donor and guide plasmids, respectively. Plates were incubated at 37 °C overnight. Colonies that grew on LB agar, but not on LB agar containing antibiotics, were picked into 5 mL LB and grown overnight at 37 °C with shaking. An aliquot of the culture (750 µL) was diluted with 750 µL of sterile 50% glycerol, frozen, and maintained at −70 °C. Cells were harvested from the remaining culture by centrifugation at 4,500*×g* for 5 min at room temperature and used for gDNA purification as described above. Whole-genome sequencing of the parental Δ*pdxB*::*kanR E. coli* BW25113 and the edited strains was carried out at SeqCenter and SeqCoast to ensure correct editing and the absence of unintended mutations elsewhere in the genome.

## Supplementary Material

msaf193_Supplementary_Data

## Data Availability

The data underlying this article are available in the article and in its online Supplementary material.
