# Supplementary material for: Mutations Elevate an Underground Pathway to a Physiologically Relevant Protopathway
Source: Mol Biol Evol. 2025 Aug 7;42(8):msaf193. doi: 10.1093/molbev/msaf193 (PMC12393044; doi:10.1093/molbev/msaf193)
Supplement: msaf193_Supplementary_Data [file msaf193_supplementary_data.zip › Supplementary Materials.pdf]

Supplementary Materials for

Mutations elevate an underground pathway to a physiologically relevant protopathway

Karl A. Widney[1,3], Lauren C. Phillips[2,3], Leo M. Rusch[2,3] and Shelley D. Copley[2,3]

[1] Department of Biochemistry, University of Colorado Boulder, Boulder, CO, 80309, USA

[2] Department of Molecular, Cellular and Developmental Biology, University of Colorado Boulder, Boulder, CO, 80309, USA

[3] Cooperative Institute for Research in Environmental Sciences, University of Colorado, Boulder, CO, 80205, USA

*Corresponding author

Email: shelley.copley@colorado.edu

Contents

Figs. S1-S6

Tables S1-S7

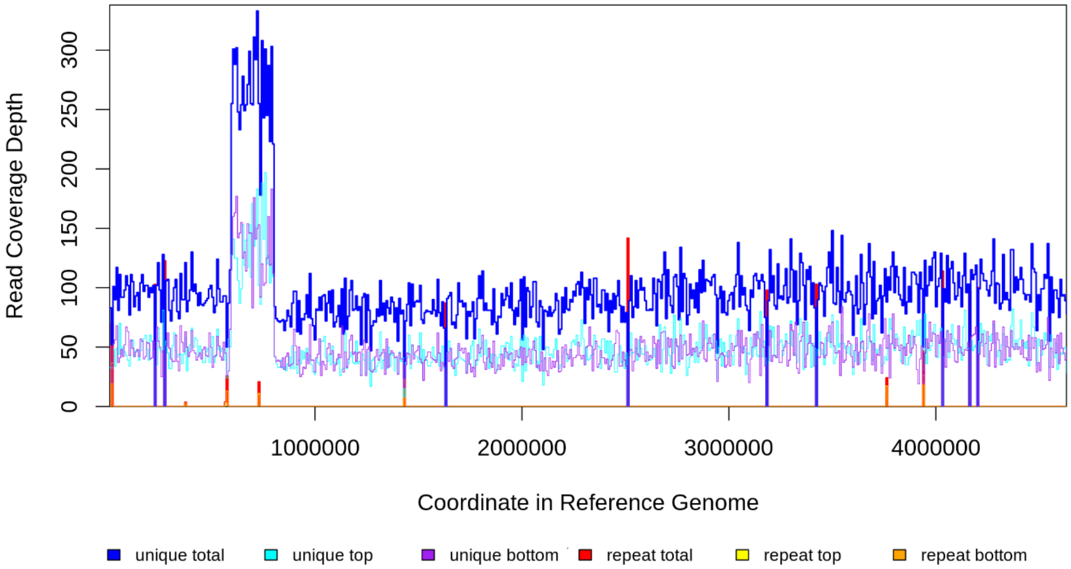

Fig. S1. Read coverage in population genomic DNA at 63 population doublings when the clone containing the amplification dominated the population. Read coverage averages 92 but is elevated to ~275 between genome coordinates 592434 and 798903.

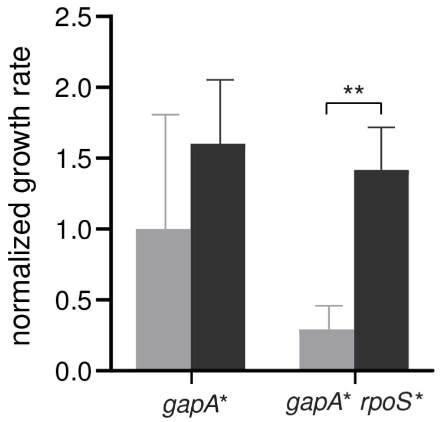

Fig. S2. Spent medium collected after growth of Δ*pdxB E. coli* improves growth of the *gapA* rpoS** strain. Light grey, growth rate in M9/glucose; dark grey, growth rate in medium collected after growth of Δ*pdxB E. coli* to OD_{600} = 0.15 in M9/glucose. Error bars represent 1 standard deviation. P-values adjusted by Dunnett T3 correction for multiple comparisons (Graphpad Prism 8). **, p-adj < 0.01.

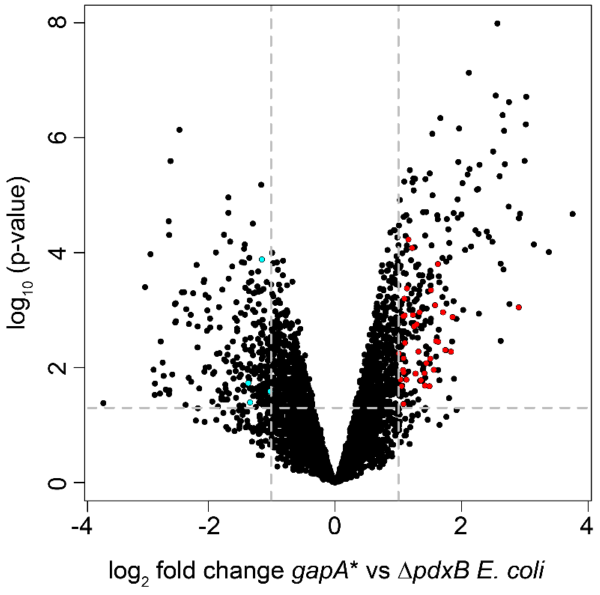

Fig. S3. The *gapA** mutation in the Δ*pdxB* background causes significant changes in gene expression in M9/glucose. Dark red, genes in the RpoS regulon that are significantly upregulated; cyan, genes in the RpoS regulon that are significantly downregulated. Horizontal grey dashed line, cutoff for statistical significance with p-adj < 0.05. Vertical grey dashed lines, cutoff for changes in expression greater than 2-fold.

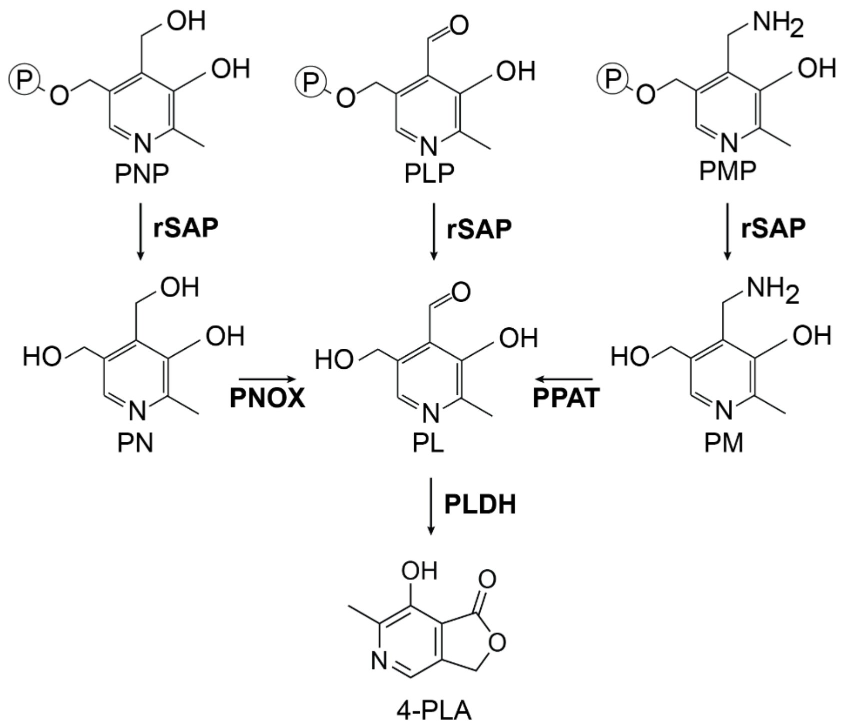

Fig. S4. Enzymes used to convert B_6 vitamers to 4-pyridoxolactone (4-PLA). rSAP, recombinant shrimp alkaline phosphatase; PNOX, pyridoxine oxidase; PPAT, pyridoxamine-pyruvate aminotransferase; PLDH, pyridoxal 4-dehydrogenase; PN, pyridoxine; PL, pyridoxal; PM, pyridoxamine.

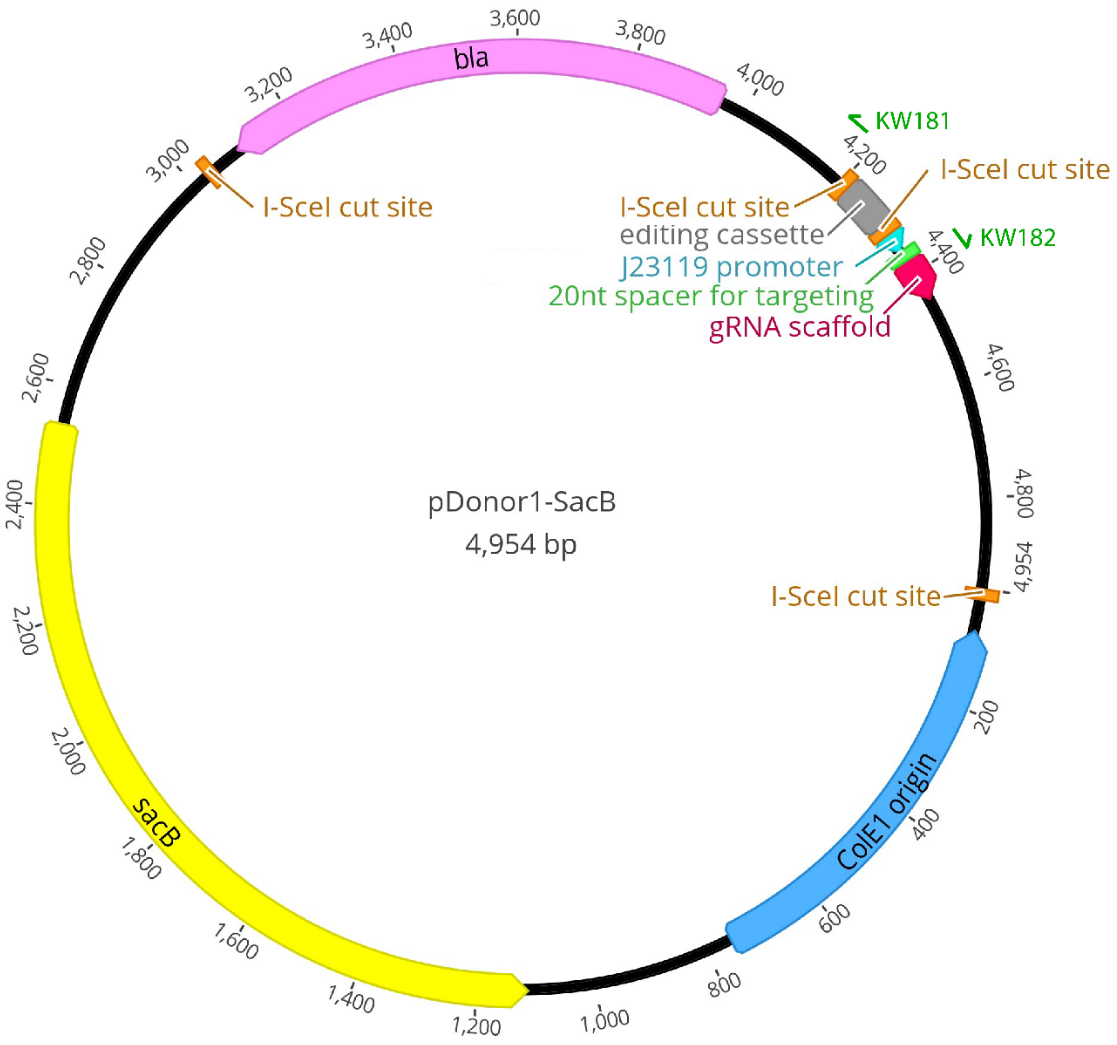

Fig. S5. Structure of the pDonor1-SacB plasmids used for one-step editing and the first round of scarless genome editing. Pink, *bla*, which encodes beta-lactamase, confers resistance to 100 μg/ml ampicillin; yellow, *sacB*, which encodes levansucrase; orange, I-SceI recognition sites; grey, landing pad for editing cassette; cyan, constitutive J23119 promoter; green, N20 segment of gRNA attacking wild-type sequences; red, scaffold segment of the sgRNA; green arrows, primers for amplifying backbone.

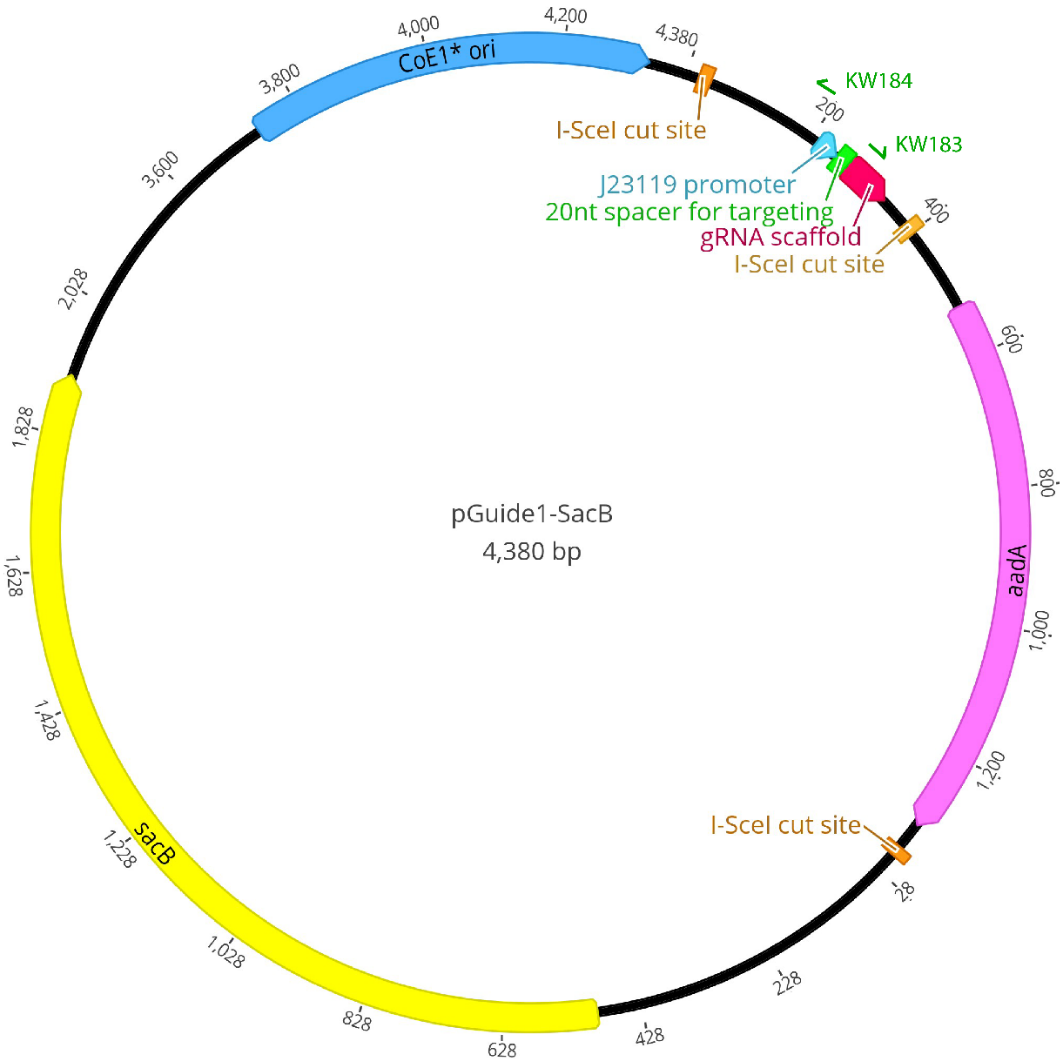

Fig. S6. Structure of the pGuide1-SacB plasmids used for the second round of scarless genome editing. Pink, *aadA*, which encodes aminoglycoside adenylyltransferase, confers resistance to 100 μg/ml spectinomycin or streptomycin; yellow, *sacB*, which encodes levansucrase; orange, I-SceI recognition sites; cyan, constitutive J23119 promoter; green, N20 segment of gRNA attacking wild-type sequences; red, scaffold segment of the sgRNA; green arrows, primers for amplifying backbone.

Table S1. Growth rates of mutant strains in M9/glucose in the absence and presence of 10 µM pyridoxal.

Strain	M9/glucose<br><br>µ<br>(h^{-1})	M9/glucose + 10 µM pyridoxal<br>µ<br>(h^{-1})
Δ*pdxB*	0.01 ± 0.01	0.43 ± 0.04
Δ*pdxB gapA**	0.04 ± 0.02	0.10 ± 0.02
Δ*pdxB rpoS**	0.01 ± 0.01	0.41 ± 0.05
Δ*pdxB* Δ3.8 kb	0.01 ± 0.01	0.39 ± 0.03
Δ*pdxB rpoC**	0.01 ± 0.01	0.65 ± 0.05
Δ*pdxB gapA* rpoS**	0.01 ± 0.01	0.12 ± 0.03
Δ*pdxB gapA** Δ3.8 kb	0.13 ± 0.05	0.11 ± 0.02
Δ*pdxB gapA* rpoC**	0.01 ± 0.01	0.30 ± 0.02
Δ*pdxB rpoS** Δ3.8 kb	0.02 ± 0.01	0.40 ± 0.02
Δ*pdxB rpoS* rpoC**	0.02 ± 0.01	0.68 ± 0.01
Δ*pdxB* Δ3.8 kb *rpoC**	0.01 ± 0.01	0.56 ± 0.02
Δ*pdxB gapA* rpoS** Δ3.8 kb	0.10 ± 0.01	0.09 ± 0.03
Δ*pdxB gapA** Δ3.8 kb *rpoC**	0.30 ± 0.02	0.26 ± 0.02
Δ*pdxB gapA* rpoS* rpoC**	0.02 ± 0.01	0.31 ± 0.01
Δ*pdxB rpoS**Δ3.8 kb *rpoC**	0.02 ± 0.01	0.57 ± 0.04
Δ*pdxB gapA* rpoS**Δ3.8 kb *rpoC**	0.32 ± 0.02	0.26 ± 0.03

Table S2. Strains used in this work.

strain	genotype	notes
E. coli DH5α	*Δ(argF-lac)*169, *φ80dlacZ58(M15), ΔphoA8, glnX44(AS), λ-, deoR481, rfbC1, gyrA96(NalR), recA1, endA1, thiE1, hsdR17*	Coli Genetic Stock Center (CGCS) strain # 14231
E. coli BW25113 *pdxB*:*kanR*	BW25113 (MG1655 Δ(*araDaraB*)567, *ΔlacZ4787(*::*rrnB-3), λ-, rph-1, Δ(rhaD-rhaB)568, hsdR514*); *pdxB* replaced with *kanR*	from the Keio collection (1)
E. coli NiCo21(DE3)	*can*::*CBD fhuA2 [lon] ompT gal* (λ DE3) *[dcm] arnA*::*CBD slyD*::*CBD glmS6Ala ΔhsdS* λ DE3 = λ *sBamHIo ΔEcoRI-B int*::*(lacI*::*PlacUV5*::*T7 gene1) i21 Δnin5*	(2)
KW274	*E. coli* BW25113 *pdxB*::*kanR*	contains the helper plasmid pDY118A; starting strain for constructing strains used for the fitness landscape; derived from the Keio collection
KW462	*E. coli* BW25113 *pdxB*::*kanR gapA**	
KW463	*E. coli* BW25113 *pdxB*::*kanR* Δ	
KW464	*E. coli* BW25113 *pdxB*::*kanR rpoC**	
KW465	*E. coli* BW25113 *pdxB*::*kanR rpoS**	
KW466	*E. coli* BW25113 *pdxB*::*kanR gapA** Δ	
KW467	*E. coli* BW25113 *pdxB*::*kanR gapA* rpoC**	
KW468	*E. coli* BW25113 *pdxB*::*kanR gapA* rpoS**	
KW470	*E. coli* BW25113 *pdxB*::*kanR* Δ *rpoS**	
KW471	*E. coli* BW25113 *pdxB*::*kanR rpoC* rpoS**	
KW472	*E. coli* BW25113 *pdxB*::*kanR gapA** Δ *rpoC**	
KW473	*E. coli* BW25113 *pdxB*::*kanR gapA** Δ *rpoS**	
KW475	*E. coli* BW25113 *pdxB*::*kanR Δ rpoC* rpoS**	
KW476	*E. coli* BW25113 *pdxB*::*kanR gapA** Δ *rpoC* rpoS** (JK1)	
KW490	*E. coli* BW25113 *pdxB*::*kanR gapA* rpoC* rpoS**	
KW492	*E. coli* BW25113 *pdxB*::*kanR* Δ *rpoC**	

Table S3. Plasmids used in this work.

plasmid	use/description	source/ref
pDY118A	helper plasmid for Cas9-assisted editing; encodes Cas9 under control of the Tet promoter, lambda Red enzymes under control of a heat-inducible promoter, I-Sce-I under control of the lacIq promoter, and chloramphenicol resistance; pSC101 temperature-sensitive origin of replication	(3)
pDonor1	plasmid encoding an editing cassette and Cas9 sgRNA under control of the constitutive J23119 promoter; confers ampicillin resistance	(3)
pDonor2	plasmid encoding an editing cassette and Cas9 sgRNA under control of the constitutive J23119 promoter; confers streptomycin resistance	(3)
pDonor1-SacB	plasmid encoding an editing cassette and Cas9 sgRNA under control of the constitutive J23119 promoter); confers ampicillin resistance; identical to pDonor1 except that it contains four I-SceI recognition sites and a gene encoding SacB to aid in plasmid curing; used as template for amplifying the backbone of donor/guide plasmids	this work
pGuide1-SacB	plasmid encoding Cas9 sgRNA under control of the constitutive J23119 promoter); confers streptomycin resistance; lacks the editing cassette in pDonor2 and contains three I-SceI recognition sites and a gene encoding SacB to aid in plasmid curing; used as template for amplifying the backbone of guide plasmids	this work
pHGDH	plasmid encoding D-2-hydroxyglutarate dehydrogenase with an N-terminal His_6-tag under control of the lac promoter; confers kanamycin resistance; used to amplify the backbone for pKAW110 and pKAW111	generous gift from Gregory Grant
pAM078	expression of ArgC with N-terminal 10xHis-tag + SUMO-tag under control of lac promoter; pET28 backbone; confers kanamycin resistance; used as template for pKAW077	(4)
pGro7	expression of the GroEL and GroES chaperones under control of the araBp promoter; pACYC origin of replication; confers chloramphenicol resistance	Takara
pHA1887	1887 bp fragment of pUC19 that contains the replication of origin and confers ampicillin resistance; used as template for backbone of pKAW0018	(5)
pT2SCb	template plasmid for amplification of an editing cassette including two transcription terminators and an I-SceI recognition site; confers chloramphenicol resistance	(5)
pKAW043	donor plasmid for introduction of synonymous codons into *gapA*; Cas9 sgRNA that targets *gapA* under control of the J23119 promoter; editing cassette contains synonymous codons; ColE1 origin of replication; contains two I-SceI recognition sites and a gene encoding SacB to aid in curing of the plasmid; confers ampicillin resistance	this work

pKAW044	donor plasmid for introduction of synonymous codons into *rpoC*; Cas9 sgRNA under control of theJ23119 promoter that targets *rpoC*; editing cassette contains synonymous codons; ColE1 origin of replication; contains two I-SceI recognition sites and a gene encoding SacB to aid in curing of the plasmid; confers ampicillin resistance	this work
pKAW045	guide plasmid for introduction of *gapA**; Cas9 sgRNA that targets *gapA* synonymous codons under control of the J23119 promoter; ColE1 origin of replication; contains two I-SceI recognition sites and a gene encoding SacB to aid in curing of the plasmid; confers streptomycin resistance	this work
pKAW046	guide plasmid for introduction of *rpoC**; Cas9 sgRNA that targets *rpoC* synonymous codons under control of the J23119 promoter; ColE1 origin of replication; contains two I-SceI recognition sites and a gene encoding SacB to aid in curing of the plasmid; confers streptomycin resistance	this work
pKAW065	guide plasmid for introduction of the 3812 bp deletion; Cas9 sgRNA that targets *ybhA* under control of the J23119 promoter; contains two I-SceI recognition sites and a gene encoding SacB to aid in curing of the plasmid; confers ampicillin resistance	this work
pKAW066	encodes linear editing cassette for I-SceI-assisted editing of *rpoS*; editing cassette is comprised of three segments: 1) a segment containing a homology arm (HA) upstream of the edit, the edit and a short downstream HA; 2) a segment containing an I-SceI recognition site and a streptomycin resistance gene; and 3) a final segment containing a short HA upstream of the edit, the edit and a downstream HA; ampicillin resistance; pMB1 origin of replication	this work
pKAW077	expression of PLDH with N-terminal 10xHis-tag under control of the lac promoter; pET28 backbone; kanamycin resistance	this work
pKAW110	expression of PPAT with N-terminal 6xHis-tag under control of the lac promoter; pET28 backbone; kanamycin resistance	this work
pKAW111	expression of PNOX with N-terminal 6xHis-tag under control of the lac promoter; pET28 backbone; kanamycin resistance	this work

Table S4. Primers used in this work.

name	sequence	use
KW182	CCAGAAATCATCCTTAGCGAA	amplification of pDonor1 backbone
KW195	ACTAGTATTATACCTAGGACTGAGC	
KW256	GTTTTAGAGCTAGAAATAGCAAGTTAAAATAAGGCTAGTCCG	amplification of pDonor2 backbone
KW181	CCAGAAATCATCCTTAGCGAA	
pHAseqF	TATCAGGGTTATTGTCTCATGAGCG	sequencing primers to verify construction of donor and guide plasmids
pHAseqR	ACTTGAGCGTCGATTTTTGTGATGC	
KW181	CCAGAAATCATCCTTAGCGAA	amplification of pDonor1-SacB
KW182	GTTTTAGAGCTAGAAATAGCAAGTTAAA	
KW183	GTTTTAGAGCTAGAAATAGCAAGTTAAAATAAGGC	amplification of pGuide1-SacB backbone
KW184	ATCCAGCATATGCGGTGTG	
KW201	AGCGAACCCCAGAGTCCCGCTAGGGATAACAGGGTAATAATGAAGTTTTAAATCAATCTAAAGTATATATG	amplification of backbones of pDonor1 and pDonor2; purple, extensions to add I-SceI recognition sites; blue, 20-nt overhang for Gibson assembly with *sacB* fragment; red, 20-nt overhang for Gibson assembly with other half of backbone
KW200	CTCACATGTTCTTTCCTGCGTAGGGATAACAGGGTAATTTATCCCCTGATTCTGTGGATAA	
KW027	CGCAGGAAAGAACATGTG	amplification of backbones of pDonor1 and pDonor2; red, 20 nt overhang for Gibson *sacB* fragment
KW202	CCTGGTTGGCTTGGTTTCATTTTAATTTAAAAGGATCTAGGTGAAGATC	
KW045	ATGAAACCAAGCCAACCAGG	amplification of *sacB*
KW063	GCGGGACTCTGGGGTTCG	
KW168	CACATTGGTCCCTGCCCGA	amplification of editing cassette from JK1 gDNA for the 3812 bp deletion
KW330	ACCGCACAAAAGGGGAGTGC	
KW170	CATTGGTGGAACCCGTTATA	screening and sequencing colonies after introduction of the 3812 bp deletion
KW331	ATAGCTAATACCGGCATCTTTC	
KW173	CAGGGCTGGCGATATAAACTGTTTG	internal primers to confirm the 3812 bp deletion
KW174	GCATTCAGGCTTCCGATGTTTCTC	

KW019	CTGGCCAGGACATCGTTTC	amplification of the *gapA** editing cassette from JK1 gDNA
KW020	ACGTCATCTTCGGTGTAGCC	
KW179	TACCGTTGAAGTGAAAGACGG	screening and Sanger sequencing of colonies after introduction of the synonymous mutations into *gapA** or the *gapA** edit; external to primers used to amplify the editing cassette
KW180	AGGACAGGGGAGATTTGTTC	
KW013	TGCTGCACGAACAGTGGT	amplification of the *rpoC** editing cassette from JK1 gDNA
KW014	TTTGTAGCTTTCTTTAGTACGACCGA	
KW198	AGGACCTGGTGGTTACCGAA	screening and Sanger sequencing of colonies after introduction of the synonymous mutations into *rpoC* or the *rpoC** edit; external to primers used to amplify the editing cassette
KW199	GCGTACAAAACCGCTTACTTC	
KW027	CGCAGGAAAGAACATGTG	amplification of the backbone for pKAW066 intermediate
KW028	AAGGGCCTCGTGATACG	
KW107	AGGCGTATCACGAGGCCCTTCACTTGGTTCATGGTCCAGCTTATGG	amplification of the upstream homology arm for pKAW066 intermediate; blue, 20-nt overhang for Gibson assembly with plasmid backbone; red, 20-nt overhang for Gibson assembly with chloramphenicol resistance cassette
KW056	ACCGCTGCCACTCTTGAGATGGCCTTAGTAGAACAGGAACCCAGTG	
KW015	ATCTCAAGAGTGGCAGC	amplification of the chloramphenicol resistance cassette for pKAW066 intermediate
KW016	TTATGCACCTCCTTGCC	
KW057	AGTGGCAGGGCGGGGCGTAATTATCACTGGGTTCCTGTTCTACTAAGG	amplification of the downstream homology arm for pKAW066 intermediate; red, 20-nt overhang for Gibson assembly with chloramphenicol resistance cassette; blue, 20-nt overhang for Gibson assembly with plasmid backbone
KW108	CTCACATGTTCTTTCCTGCGCAGTACATCAACCAGTACGCCTATCT	
KW334	TTATCACTGGGTTCCTTGTTCTACTAA	amplification of the backbone from pKAW066 intermediate
KW335	TTTAGCTTCCTTAGCTCCTGAAAATC	
KW332	CAGGAGCTAAGGAAGCTAAAATGAGGGAAGCGGTGATCGC	amplification of *smR*; red and blue, 20-nt overhangs for Gibson assembly with pKAW066 intermediate backbone
KW333	AACAAGGAACCCAGTGATAATTATTTGCCGACTACCTTGGTGATCTCG	

KW059	ACCAATCTCACCAAGGTAAAGC	amplification of the *rpoS** editing cassette from pKAW066
KW385	TAGCACCGGAACCAGTTCAACAC	
KW061	CACTTGGTTCATGGTCCAGCTTA	screening and Sanger sequencing of colonies after introduction of the *rpoS** edit; external to primers used to amplify the editing cassette
KW062	CAGTACATCAACCAGTACGCCTA	
KW199	GCGTACAAAACCGCTTACTTC	
KW418	ACGCTGGAATGGTACGCCACTGAGATCCGGCTG CTAACAAAGC	amplification of the backbone of pAM078 for pKAW077; blue, 20-nt overhang for Gibson assembly with end of *pldh* gene; red, 20-nt overhang for Gibson assembly with beginning of *pldh* gene
KW419	TTCCCTGCAAGACGTTCAGTGCCGCTGCTGTGA TGATGATG	
KW234	ACCTGCAGCCAAGCTTGCG	amplification of the backbone of pHGDH; used for Gibson assembly of pKAW110 and pKAW111
KW235	GGATCCCTGGAAGTACAGGTTTTCATGG	
KW305	GCTAGTTATTGCTCAGCGG	screening and Sanger sequencing of gene inserts into the pAM078 and pHGDH backbones
KW246	GCGAAATTAATACGACTCACTATAGG	

Table S5. Oligonucleotides for insertion of gRNA sequences into guide plasmids. Complementary oligonucleotides were annealed and then inserted into plasmids by Gibson assembly. Green, 20 nt gRNA spacer; blue and purple, overhangs for Gibson assembly; black, J23119 promoter.

gRNA target	sequence	plasmid
gapA	GTATTTCACACCGCATATGCTGGATTTGACAGCTAGCTCAGTCCTAGGTATAATACT AGTCAGTACTTTACCTACAGCTTGTTTTAGAGCTAGAAATAGCAAGTT	pKAW045
	AACTTGCTATTTCTAGCTCTAAAACAAGCTGTAGGTAAAGTACTGACTAGTATTATA CCTAGGACTGAGCTAGCTGTCAAATCCAGCATATGCGGTGTGAAATAC	
rpoC	GTATTTCACACCGCATATGCTGGATTTGACAGCTAGCTCAGTCCTAGGTATAATACT AGTGAACGTACGCATAGTAAGTTGTTTTAGAGCTAGAAATAGCAAGTT	pKAW046
	AACTTGCTATTTCTAGCTCTAAAACAACTTACTATGCGTACGTTCACTAGTATTATA CCTAGGACTGAGCTAGCTGTCAAATCCAGCATATGCGGTGTGAAATAC	
ybhA	CGTAGGGATAACAGGGTAATTTGACAGCTAGCTCAGTCCTAGGTATAATACTAGTCG TCAGGGCGAACTTCCATAGTTTTAGAGCTAGAAATAGC	pKAW065
	GCTATTTCTAGCTCTAAAACTATGGAAGTTCGCCCTGACGCACTAGTATTATACCTA GGACTGAGCTAGCTGTCAAATTACCCTGTTATCCCTACG	

Table S6. gBlocks used for first round of genome editing. Each gBlock contains gRNA expression cassette and editing cassette sequences flanked by 20 nt extensions for Gibson assembly into a plasmid backbone. Blue, upstream Gibson assembly extension; bold, synonymous codons; magenta, J23119 constitutive promoter; purple, 20 nt gRNA spacer; red, downstream Gibson assembly extension.

first round of *gapA* editing	TCGCTAAGGATGATTTCTGGAACGCTTCCTGCACCACCAACTGCCTGGCTCCGCTGGCTAAAG<br>TTATCAACGATAACTTCGGCATCATCGAAGGTCTGATGACCACCGTTCACGCTACTACCGCTA<br>CTCAGAAAACCGTTGATGGCCCGTCTCACAAAGACTGGCGCGGCGGCCGCGGCGCTTCCCAGA<br>ACATCATCCCG**AGT**TC**A**AC**T**GG**C**GC**A**GC**C**AAAGCTGTAGGTAAAGTACTGCCAGAACTGAATG<br>GCAAACTGACTGGTATGGCGTTCCGCGTTCCGACCCCGAACGTATCTGTAGTTGACCTGACCG<br>TTCGTCTGGAAAAAGCTGCAACTTACGAGCAGATCAAAGCTGCCGTTAAAGCTGCTGCTGAAG<br>GCGAAATGAAAGGCGTTCTGGGTTGACAGCTAGCTCAGTCCTAGGTATAATACTAGTACCTAC<br>AGCTTTAGCAGCACGTTTTAGAGCTAGAAATAGC
first round of *rpoC* editing	TCGCTAAGGATGATTTCTGGAACAGTGGTGTGACCTGCTGGAAGAGAACTCTGTCGACGCGGT<br>TAAAGTACGTTCTGTTGTATCTTGTGACACCGACTTTGGTGTATGTGCGCACTGCTACGGTCG<br>TGACCTGGCGCGTGGCCACATCATCAACAAGGGTGAAGCAATCGGTGTTATCGCGGCACAGTC<br>CATCGGTGAACC**A**GG**C**AC**C**CA**A**CT**T**AC**T**ATGCGTACGTTCCACATCGGTGGTGCGGCATCTCG<br>TGCGGCTGCTGAATCCAGCATCCAAGTGAAAAACAAAGGTAGCATCAAGCTCAGCAACGTGAA<br>GTCGGTTGTGAACTCCAGCGGTAAACTGGTTATCACTTCCCGTAATACTGAACTGAAACTGAT<br>CGACGAATTCGGTCGTACTAAATTGACAGCTAGCTCAGTCCTAGGTATAATACTAGTACCGAT<br>GTGGAACGTACGCAGTTTTAGAGCTAGAAATAGC

Table S7. gBlocks containing genes used for protein expression. In some cases, extensions were added for Gibson Assembly. Blue, upstream extension; red, downstream extension.

gene	sequence
Mesorhizobium loti pyridoxal 4-dehydrogenase (PLDH)	ACTGAACGTCTTGCAGGGAAAACTGCGCTTGTTACAGGAGCGGCACAGGGCATCGGCAA<br>GGCGATTGCAGCTCGCCTTGCTGCAGATGGAGCGACAGTGATCGTGAGTGATATCAATG<br>CGGAAGGTGCGAAGGCTGCTGCCGCGTCGATTGGGAAAAAGGCTCGCGCAATCGCTGCA<br>GACATCTCTGATCCAGGATCAGTCAAGGCGCTGTTCGCCGAAATCCAGGCTCTTACCGG<br>CGGTATTGACATTCTGGTCAACAATGCCTCTATTGTACCATTTGTAGCCTGGGACGACG<br>TAGATTTAGACCATTGGCGCAAGATCATTGATGTGAATTTGACAGGCACGTTTATTGTT<br>ACCCGTGCCGGCACCGACCAGATGCGTGCGGCAGGAAAAGCCGGTCGTGTGATCTCTAT<br>TGCATCTAACACATTCTTTGCCGGCACTCCTAATATGGCGGCCTATGTGGCTGCGAAGG<br>GTGGGGTTATCGGATTTACTCGCGCATTAGCGACAGAGTTAGGTAAGTATAATATTACC<br>GCAAACGCGGTTACTCCAGGTTTAATCGAGAGCGACGGGGTGAAAGCGAGCCCCCATAA<br>TGAAGCCTTCGGATTTGTTGAGATGCTTCAAGCGATGAAGGGCAAGGGGCAACCGGAAC<br>ATATTGCGGACGTCGTTTCATTCCTTGCTTCGGACGATGCGCGTTGGATTACTGGTCAA<br>ACCCTTAATGTCGACGCTGGAATGGTACGCCAC
Mesorhizobium loti pyridoxamine-pyruvate amino-transferase (PPAT)	ACCTGTACTTCCAGGGATCCCGTTACCCTGAGCATGCGGACCCCGTTATCACACTGACT<br>GCTGGCCCCGTCAATGCTTACCCCGAGGTTTTACGCGGCCTGGGTCGTACGGTACTGTA<br>TGACTACGACCCTGCGTTCCAATTGCTGTATGAGAAAGTCGTCGATAAAGCTCAGAAGG<br>CAATGCGCCTGTCAAATAAACCTGTAATTCTGCATGGCGAGCCCGTCCTGGGATTAGAA<br>GCGGCGGCGGCTTCGTTGATTTCTCCTGACGATGTAGTGTTAAACCTGGCTAGTGGCGT<br>TTACGGCAAGGGTTTTGGTTACTGGGCAAAACGCTACTCACCCCATTTGTTAGAAATCG<br>AGGTCCCATACAACGAGGCGATTGACCCGCAAGCTGTTGCCGATATGCTTAAAGCACAC<br>CCCGAGATCACCGTTGTATCAGTCTGCCACCACGACACGCCCAGCGGAACCATTAATCC<br>TATCGATGCGATTGGGGCCTTGGTTTCAGCCCACGGCGCTTACCTGATTGTGGACGCAG<br>TGAGTAGTTTCGGGGGTATGAAGACTCATCCTGAAGACTGTAAAGCTGACATTTATGTG<br>ACCGGCCCGAACAAGTGCCTTGGGGCACCCCCCGGATTGACTATGATGGGTGTTTCGGA<br>GCGTGCATGGGCGAAAATGAAGGCCAATCCCTTAGCTCCACGCGCAAGCATGTTATCCA<br>TCGTTGATTGGGAGAACGCATGGTCGCGCGATAAGCCGTTTCCTTTTACGCCGAGCGTG<br>TCCGAAATTAACGGATTAGACGTGGCGCTGGATTTATATTTAAATGAGGGTCCCGAAGC<br>AGTTTGGGCACGCCATGCCTTAACAGCGAAGGCGATGCGCGCGGGCGTGACCGCTATGG<br>GATTGTCAGTCTGGGCTGCGTCAGACTCAATCGCTAGTCCCACTACTACAGCTGTGCGC<br>ACGCCCGATGGGGTGGACGAAAAGGCTTTACGTCAGGCAGCACGTGCTCGTTATGGTGT<br>TGTGTTCTCTTCAGGCCGCGGAGAAACATTAGGCAAACTTACTCGTATCGGACACATGG<br>GTCCTACCGCACAGCCCATTTATGCGATTGCAGCATTAACGGCACTGGGAGGCGCAATG<br>AACGCCGCGGGCCGCAAACTGGCAATCGGAAAAGGTATTGAAGCGGCGCTTGCGGTCAT<br>CGACGCAGACGCTTGAACCTGCAGCCAAGCTTGCGG
Mesorhizobium loti pyridoxine oxidase (PNOX)	ACCTGTACTTCCAGGGATCCACTCGTGCGAAAGTAGAACATGCACCCAATTGTGATATC<br>GTGATTGTGGGGGGGGGATCTGCGGGCTCCTTGTTAGCGGCCCGTCTTTCGGAGGACCC<br>GGATTCTCGTGTTTTGCTTATTGAAGCGGGCGAGGAACCCACTGATCCCGATATCTGGA<br>ACCCCGCAGCATGGCCTGCTCTTCAAGGACGTAGTTATGACTGGGATTATCGCACAGAA<br>GCGCAAGCAGGGACAGCGGGACGCGCACACCACTGGGCCCGTGGTCGCCTTATTGGGGG<br>GTCCAGCTGCTTACATGCAATGGGCTATATGCGCGGACATCCATCTGACTTTCAAGCAT<br>GGGTAGATGCCTCCGGGGATCGCCGCTGGGGTTGGGATGAACTTCTGCCCGTGTTTCAA<br>GCGATTGAAGACCATCCACTGGGTGGAGATGGAATCCATGGAAAAGGCGGACCCTTACC

	TATTCATTTGCCCGCGGATGAAGTTAGTCCACTTGCACGTGCTTTTATTGAAGCAGGCG<br>CCTCTCTTGGACTTCCACGCCTGGAAGGCCACAATTCGGGAGAGATGATCGGGGTCACT<br>CCGAACTCTTTGAACATTCGTGATGGTCGCCGTGTCACAGCAGCGGACGCGTGGTTGAC<br>CAAGGCGGTACGCGGGCGTAAGAATCTTACCATCTTGACAGGGAGCCGTGTGCGCCGCT<br>TGAAGTTAGAGGGTAATCAAGTCCGTAGTTTAGAAGTAGTGGGTCGTCAGGGAAGTGCT<br>GAAGTCTTTGCGGATCAGATTGTCCTGTGTGCGGGAGCTTTAGAGTCACCTGCCTTATT<br>GATGCGCAGCGGTATCGGACCCCATGACGTACTGGATGCCGCCGGTGTGGGGTGCTTAA<br>TTGACATGCCAGATATCGGACGCAACCTGCAAGATCACTTACTTGGGGCTGGTAATTTG<br>TACGCAGCACGCAAACCAGTCCCGCCAAGCCGTTTGCAGCATAGCGAAAGTATGGCGTA<br>CATGCGCGCCGATAGTTTTACGGCGGCCGGACAGCCAGAAATCGTAGTCGGGTGCGGTG<br>TCGCTCCTATCGTTTCAGAATCGTTTCCGGCTCCTGCGGCCGGGTCTGCCTATAGTTTA<br>TTGTTTGGAATCACCCACCCGACTAGTCGCGGAAGCGTTCGCATCTCCGGTCCTGAGCT<br>TGGTGATCGTCTTATCATTGATCCTGCGTACTTGCAGACGGGGCGTGATCGCGAGCGTT<br>TCCGCCGTGCACTGGAGGCTTCCCGTACTATCGGTCACCGCGACGAATTAGCGGGGTGG<br>CGTGAACGTGAACTTCTTCCGGGGACGCCCAACTCCGCAGCAGAAATGGACGACTTCAT<br>TGCTCGCTCTGTAATTACTCACCATCACCCCTGCGGTACGTGTCGTATGGGAAAAGACC<br>CGGATGCTGTCGTAGACGCGAATTTACGTTTAAAGGCTCTGGACAACTTATTCGTAGTT<br>GATGCGAGCATTATGCCAAATTTGACCGCAGGCCCCATTCACGCAGCTGTACTTGCTAT<br>TGCTGAGACGTTTGCCCGCCAGTATTAAACCTGCAGCCAAGCTTGCGG

Table S8. Fragments used to construct donor and guide plasmids for genome editing by Gibson assembly.

plasmid	purpose	fragment 1			fragment 2			fragment 3			fragment 4		
		content	source	primers	content	source	primers	content	source	primers	content	source	primers
pDonor1-SacB	template for first-round editing plasmids	*ampR*, editing cassette, sgRNA	pDonor2	KW200 + KW201	ColE1, *oriV*	pDonor2	KW027 + KW202	*sacB*	pJH110	KW045 + KW063			
pGuide1-SacB	template for second-round editing plasmids	ColE1 *oriV*	pDonor1	KW202 + KW027	sgRNA and *smR*	pDonor1	KW200 + KW203	*sacB*	pJH110	KW045 + KW063			
pKAW043	first-round *gapA* editing plasmid	sgRNA scaffold, ColE1 *oriV*, *sacB*, *ampR*	pDonor1-SacB	KW181 + KW182	editing cassette and sgRNA	gBlock from IDT							
pKAW044	first-round *rpoC* editing plasmid	sgRNA scaffold, ColE1 *oriV*, *sacB*, *ampR*	pDonor1-SacB	KW181 + KW182	editing cassette and sgRNA	gBlock from IDT							
pKAW045	second-round *gapA* editing plasmid	sgRNA scaffold, *smR*, *sacB*, ColE1 *oriV*	pGuide1-SacB	KW183 + KW184	sgRNA	two annealed oligos from IDT	KW187 + KW188						
pKAW046	second-round *rpoC* editing plasmid	sgRNA scaffold, *smR*, *sacB*, ColE1 *oriV*	pGuide1-SacB	KW183 + KW184	sgRNA	two annealed oligos from IDT	KW189 + KW190						
pKAW065	3812 bp deletion editing plasmid	sgRNA scaffold, ColE1 *oriV*, *sacB*, *ampR*	pDonor1-SacB	KW195 + KW182	sgRNA	two annealed oligos from IDT	KW189 + KW190						
pKAW066 inter-mediate	plasmid encoding the *rpoS** editing cassette	ColE1 *oriV*, *ampR*	pHA1887	KW027 + KW028	*cmR*	pT2SCb	KW015 + KW016	up-stream *rpoS* homol-ogy arm	JK1 gDNA	KW107+ KW056	down-stream *rpoS* homol-ogy arm	JK1 gDNA	KW057 + KW108
pKAW066	plasmid encoding the *rpoS** editing cassette	ColE1 *oriV*, *ampR*, *rpoS** editing cassette	pKAW066 inter-mediate	KW334 + KW335	*smR*	pDonor1	KW332 + KW333						

pKAW077	PLDH expression plasmid	10X His-tag, *lacI*, pBR322 *oriV*, *kanR*, F1 *oriV*	pAM078	KW418 + KW419	PLDH	gBlock from Twist Bio-science							
pKAW110	PPAT expression plasmid	6X His-tag, *lacI*, pBR322 *oriV*, *kanR*	pHGDH	KW234 + KW235	PPAT	gBlock from Twist Bio-science							
pKAW111	PNOX expression plasmid	6X His-tag, *lacI*, pBR322 *oriV*, *kanR*	pHGDH	KW234 + KW235	PNOX	gBlock rom Twist Bio-science							

References

1. T. Baba *et al.*, Construction of *Escherichi coli* K-12 in-frame, single-gene knock-out mutants -- the Keio collection. *Mol. Systems Biol.* **2**, Article number 2006.0008 (2006).
2. C. Robichon, J. Luo, T. B. Causey, J. S. Benner, J. C. Samuelson, Engineering *Escherichia coli* BL21(DE3) derivative strains to minimize *E. coli* protein contamination after purification by immobilized metal affinity chromatography. *Appl Environ Microbiol* **77**, 4634-4646 (2011).
3. D. D. Yang, L. M. Rusch, K. A. Widney, A. B. Morgenthaler, S. D. Copley, Synonymous edits in the *Escherichia coli* genome have substantial and condition-dependent effects on fitness. *Proc Natl Acad Sci U S A* **121**, e2316834121 (2024).
4. A. B. Morgenthaler *et al.*, Mutations that improve efficiency of a weak-link enzyme are rare compared to adaptive mutations elsewhere in the genome. *Elife* **8** (2019).
5. J. Kim, A. M. Webb, J. P. Kershner, S. Blaskowski, S. D. Copley, A versatile and highly efficient method for scarless genome editing in *Escherichia coli* and *Salmonella enterica*. *BMC Biotechnol* **14**, 84 (2014).
